# Comparative genomics analysis to differentiate metabolic and virulence gene potential in gastric versus enterohepatic *Helicobacter* species

**DOI:** 10.1186/s12864-018-5171-2

**Published:** 2018-11-20

**Authors:** Anthony Mannion, Zeli Shen, James G. Fox

**Affiliations:** Division of Comparative Medicine, Massachusetts Institute of Technology, Cambridge, MA USA

**Keywords:** Gastric and enterohepatic *Helicobacter species*, Gastrointestinal pathogens, Phylogenetic classification, Metabolism, Virulence factor genes, Comparative genome analysis

## Abstract

**Background:**

The genus *Helicobacter* are gram-negative, microaerobic, flagellated, mucus-inhabiting bacteria associated with gastrointestinal inflammation and classified as gastric or enterohepatic *Helicobacter species* (EHS) according to host species and colonization niche. While there are over 30 official species, little is known about the physiology and pathogenic mechanisms of EHS, which account for most in the genus, as well as what genetic factors differentiate gastric versus EHS, given they inhabit different hosts and colonization niches. The objective of this study was to perform a whole-genus comparative analysis of over 100 gastric versus EHS genomes in order to identify genetic determinants that distinguish these *Helicobacter species* and provide insights about their evolution/adaptation to different hosts, colonization niches, and mechanisms of virulence.

**Results:**

Whole-genome phylogeny organized *Helicobacter species* according to their presumed gastric or EHS classification. Analysis of orthologs revealed substantial heterogeneity in physiological and virulence-related genes between gastric and EHS genomes. Metabolic reconstruction predicted that unlike gastric species, EHS appear asaccharolytic and dependent on amino/organic acids to fuel metabolism. Additionally, gastric species lack de novo biosynthetic pathways for several amino acids and purines found in EHS and instead rely on environmental uptake/salvage pathways. Comparison of virulence factor genes between gastric and EHS genomes identified overlapping yet distinct profiles and included canonical cytotoxins, outer membrane proteins, secretion systems, and survival factors.

**Conclusions:**

The major differences in predicted metabolic function suggest gastric species and EHS may have evolved for survival in the nutrient-rich stomach versus the nutrient-devoid environments, respectively. Contrasting virulence factor gene profiles indicate gastric species and EHS may utilize different pathogenic mechanisms to chronically infect hosts and cause inflammation and tissue damage. The findings from this study provide new insights into the genetic differences underlying gastric versus EHS and support the need for future experimental studies to characterize these pathogens.

**Electronic supplementary material:**

The online version of this article (10.1186/s12864-018-5171-2) contains supplementary material, which is available to authorized users.

## Background

Since *Helicobacter pylori* was discovered in 1982 as the cause of chronic gastritis and later established its role in peptic ulcers and stomach cancers [1, 2], the genus *Helicobacter* has expanded to include multiple enterohepatic *Helicobacter species* (EHS) that colonize and can induce inflammation and cancer in the lower bowel, liver, and gallbladder in susceptible hosts [3–5]. The genus now includes over 30 formally named species. These gram-negative, spiral-shaped bacterial species have been detected and isolated from the stomach, gastrointestinal tract, liver, and gallbladder in mammals, birds, and reptiles.

In general, *Helicobacter species* are associated with chronic inflammation and the development of cancer; however, they often colonize their hosts as pathobionts [3, 6]. Chronic infection by *Helicobacter spp.* in immunocompetent hosts can cause subclinical disease that during immunocompromised states can manifest with overt clinical signs and pathology. Most *Helicobacter spp.* have been isolated from animal reservoirs with zoonotic potential. Additionally, experimental and spontaneous animal models have shown infection by EHS can induce gastrointestinal, hepatic, and biliary tract inflammatory pathology and cancers [3]. Most tantalizing is the possibility that, analogous to *H. pylori* infection with gastric inflammation and cancer, EHS may instigate human inflammatory bowel disease, colorectal cancer, and hepatobiliary disease [7, 8]. Nevertheless, most research has focused on *H. pylori* and related gastric species that have been isolated and cause disease in humans, leaving a void in our understanding of the mechanisms of colonization and virulence potential in EHS.

The advent of whole genome sequencing has rapidly enhanced the characterization of *Helicobacter spp.* In 1997, the first *H. pylori* genome was published [9], and today genomes from over 1,000 different strains are available. Bioinformatic analyses have provided invaluable insights about the physiology and mechanisms of virulence of *H. pylori*. Later in 2003, the genome sequence for the prototype EHS, *H. hepaticus*, was published [10]. Genomic comparison of *H. pylori* versus *H. hepaticus* revealed considerable differences in gene structure and content [10], suggesting that important distinctions underlie the contrasting colonization niches and pathogenic potentials of gastric versus EHS.

Given the different physiology and environmental conditions in the stomach versus the lower intestine, such as pH, nutrient digestion/availability, and the microbiome, we have hypothesized that prominent genetic differences evolved between gastric versus EHS. By characterizing and comparing genomes of representative gastric versus EHS, we have identified features that distinguish these different species, have provided a rationale for their adaptation to different colonization niches, and have highlighted differences in virulence potential. To complement these efforts, we have also sequenced over 30 novel EHS genomes, thereby substantially increasing the number of EHS genomes available to the research community. Identifying these similarities and differences is critical for understanding the unique physiology and pathogenic potential of current and anticipated identification of additional *Helicobacter spp.*, especially in the context of human infection and zoonotic disease.

## Results

### Phylogenetic classification of gastric and enterohepatic *Helicobacter* species

**Fig. 1:**
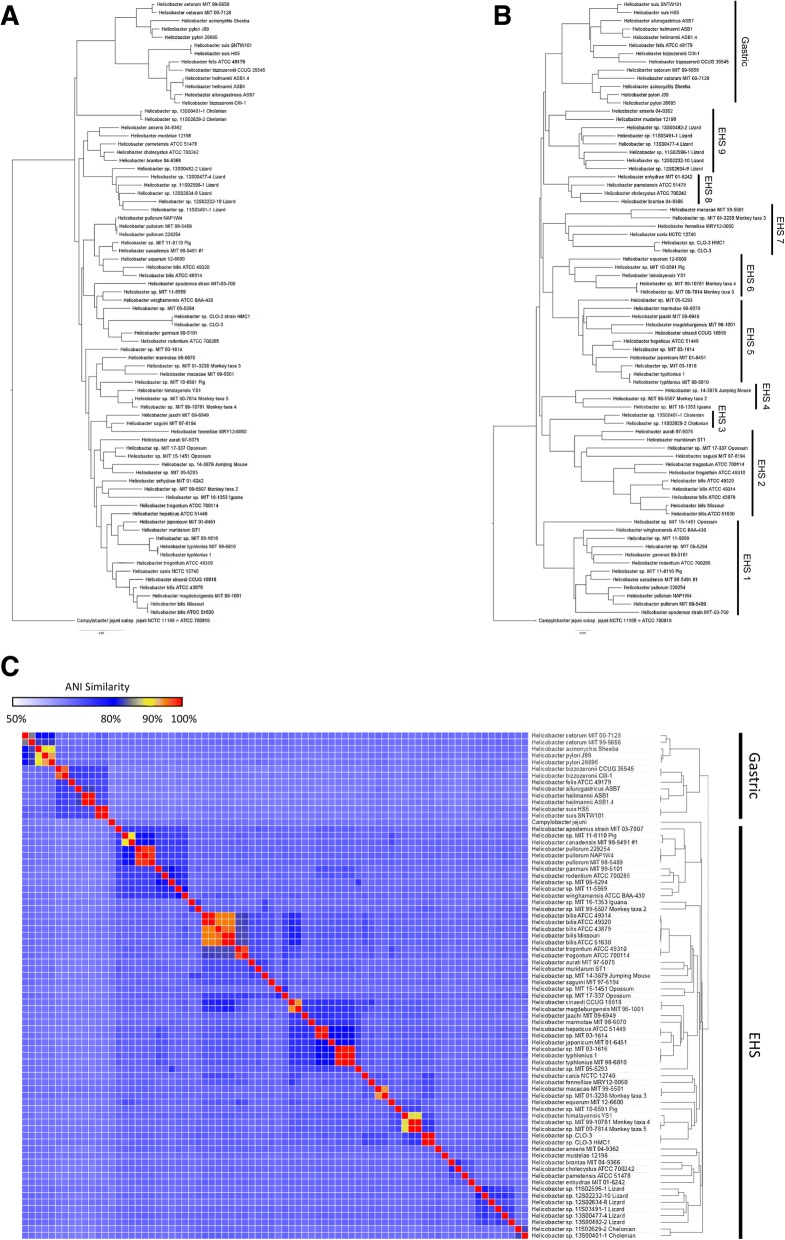
**a**) 16s rRNA gene and **b**) pan-genome phylogenetic trees both differentiated gastric and EHS genomes. The pan-genome tree more accurately organized EHS into clades consistent with known phenotypic and genetic similarities (e.g., size, morphology, biochemical traits). **c**) Heatmap and hierarchal clustering of ANI values. Genomes clustered into gastric versus EHS clades based on ANI similarity that resembled the pan-genome tree. See supplement for table with ANI values (Additional file 1: Table S1).

Phylogenetic trees based on 16s rRNA genes sequences, pan-genome orthologous gene clusters, and average nucleotide identity (ANI) similarity were constructed for taxonomic organization of gastric and EHS. Interestingly, the phylogenetic organization of species differed between trees. Based on 16s rRNA gene sequences, gastric and EHS did not always cluster with other strains and/or species in their respective subgroupings (i.e., gastric or EHS) (Fig. 1a). Previously, it has been noted that phylogenetic organization of *Helicobacter spp.* based on 16s rRNA gene sequences is discordant with phylogenies based on other genes (such as 23s rRNA or *hsp60*), isolation/colonization site, biochemical traits, or morphological characteristics [11]. However, pan-genome phylogenetic trees clearly differentiated gastric and EHS from each other (Fig. 1b). Furthermore, EHS appeared to separate into 9 clades (Fig. 1b). Hierarchical clustering of ANI, an in silico surrogate to experimental DNA-DNA hybridization [12–14], organized genomes into a dendrogram that more closely resembled the whole-genome than the 16s rRNA phylogenetic tree (Fig. 1c). An ANI threshold of ≥95% appears appropriate for differentiating gastric and EHS, although some *H. pylori* strain-strain comparisons had ANI values slightly less than 95% (Additional file 1: Table S1).

According to the phylogenetic trees, the genetic basis that determines host colonization appears more biased for anatomical niches (i.e., stomach versus lower intestine) rather than assigned to a particular host species, recognizing that in some cases, particular *Helicobacter spp.* have been isolated in only select hosts. For example, *Helicobacter spp.* isolated from reptiles fall into three different clades. Likewise, even within clades in which all species were isolated from mammals, there are different hosts, such as clade 4, which includes rodent, non-human primates, and pigs. Interestingly, *H. bilis* strains have been isolated from human, rodent, pig, dog, and sheep sources and appear to diverge into branches irrespective of their host. However, *H. mustelae* and *H. enhydrae* were notable outliers. *H. mustelae* has been traditionally classified as a gastric species because it can colonize and cause gastric disease in ferrets [15], but its genetic profile appears more similar to EHS and consequently was classified as so in subsequent analyses. *H. enhydrae* is a novel species isolated from inflamed gastric tissue of southern sea otters, a mustelid related to ferrets [16], and was also considered an EHS in this study.

Next, we sought to identify and compare the genetic determinants that differentiate the physiological and pathogenic potential of gastric species versus EHS. This included studying how *H. mustelae*, a phylogenetically-classified EHS, colonizes the stomach.

### Gastric and enterohepatic species have different genomic characteristics and gene annotations

**Fig. 2:**
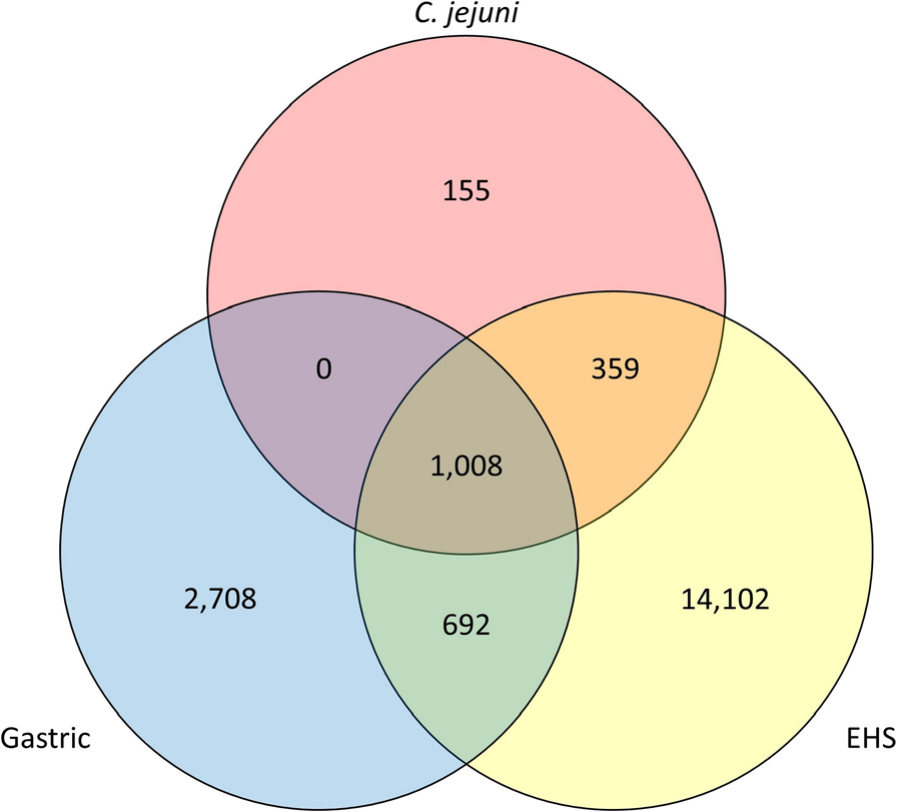
Three-way Venn diagram showing number of shared and unique orthogroups for gastric, EHS, and *C. jejuni* genomes

Comparison of gross genomic characteristics found that EHS in general have larger genome sizes with lower GC content and encode more protein coding sequences than gastric species (see Additional file 2). EHS are physically larger than gastric species which may accommodate for their larger genomes and more putative gene products. A total of 19,024 orthogroups were identified in the genomes of gastric, EHS, and *Campylobacter jejuni*, a close relative to the *Helicobacter* genus (Fig. 2). 1,008 of these were common to gastric, EHS, and *C. jejuni* genomes, while 692 were shared between just gastric and EHS genomes. 2,708 and 14,102 orthogroups were unique to gastric and EHS genomes, respectively, indicating substantial genetic diversity and heterogeneity between EHS and gastric species.

An overwhelming majority of the gastric- or EHS-specific orthogroups were hypothetical proteins (Additional file 1: Tables S2, S3). These hypothetical annotations were predicted to contain autotransportator, transmembrane, signal peptides, intracellular signaling, protein binding and/or unknown protein domains according to InterProScan analysis. Notable unique genes identified in the gastric clade included outer membrane proteins, D-amino acid dehydrogenase involved in D-phenylalanine metabolism, and lipid A 1-phosphatase (*lpxE*) functioning in lipid A modification during lipopolysaccharide (LPS) biosynthesis. Between EHS clades, hypothetical proteins were also mainly different. However, notable clade-specific genes include: a predicted oxidoreductase for secondary bile acid metabolism unique to clades 1; a hypothetical protein with an aspartic-type endopeptidase activity domain unique to clade 2; and a carbon monoxide dehydrogenase for carbon fixation and energy production from carbon monoxide unique to clade 3.

Phylogenetic organization by 16s and 23s rRNA gene similarity [11] and more recently whole-genome analysis [17] by other groups have shown that *H. mustelae* more closely groups with EHS than gastric species. To further study this, we identified the orthogroups shared or unique to *H. mustelae* compared with other EHS and gastric species (Additional file 1: Table S4). Of the 2,356 orthogroups identified in *H. mustelae*, 269 were unique to only *H. mustelae*, 179 were shared with EHS but no gastric species and only 15 orthogroups were shared between gastric species but not EHS, reinforcing that *H. mustelae* has more genetic similarity with EHS than gastric species. Almost all of the 269 orthogroups unique to *H. mustelae* encoded for hypothetical proteins aside from annotations for a membrane-fusion protein, signal-transduction regulatory protein *flgR*, penicillin-binding protein, and four different putative autotransporter protein genes. Of the orthogroups shared between *H. mustelae* and gastric species, this included several outer membrane proteins and membrane-associated transporters. Interestingly, *H. mustelae* along with the gastric species *H. acinonychis*, *H. cetorum*, and *H. felis* harbor two different urease *ureA* genes that belong to different orthogroups (*ureA*: OG0001420 versus *ureA2*: OG0004327). Unlike *ureA*, *ureA2* was not contained within the *ureBIEFGH* gene cluster, but instead was flanked by the *ureB2* gene suggesting expression of a complete urease enzyme (*ureA2B2*). Previously, *ureA2B2* has been shown to form an enzymatically active urease that is expressed only under nickel-restricted conditions [18]. The presence of two different urease gene systems in *H. mustelae* may enable this EHS-like organism to colonize the ferret stomach. Other gastric species and EHS genomes did not encode the *ureA2B2* operon; however, the presence and role of urease genes in gastric and EHS is discussed in a subsequent section.

**Fig. 3:**
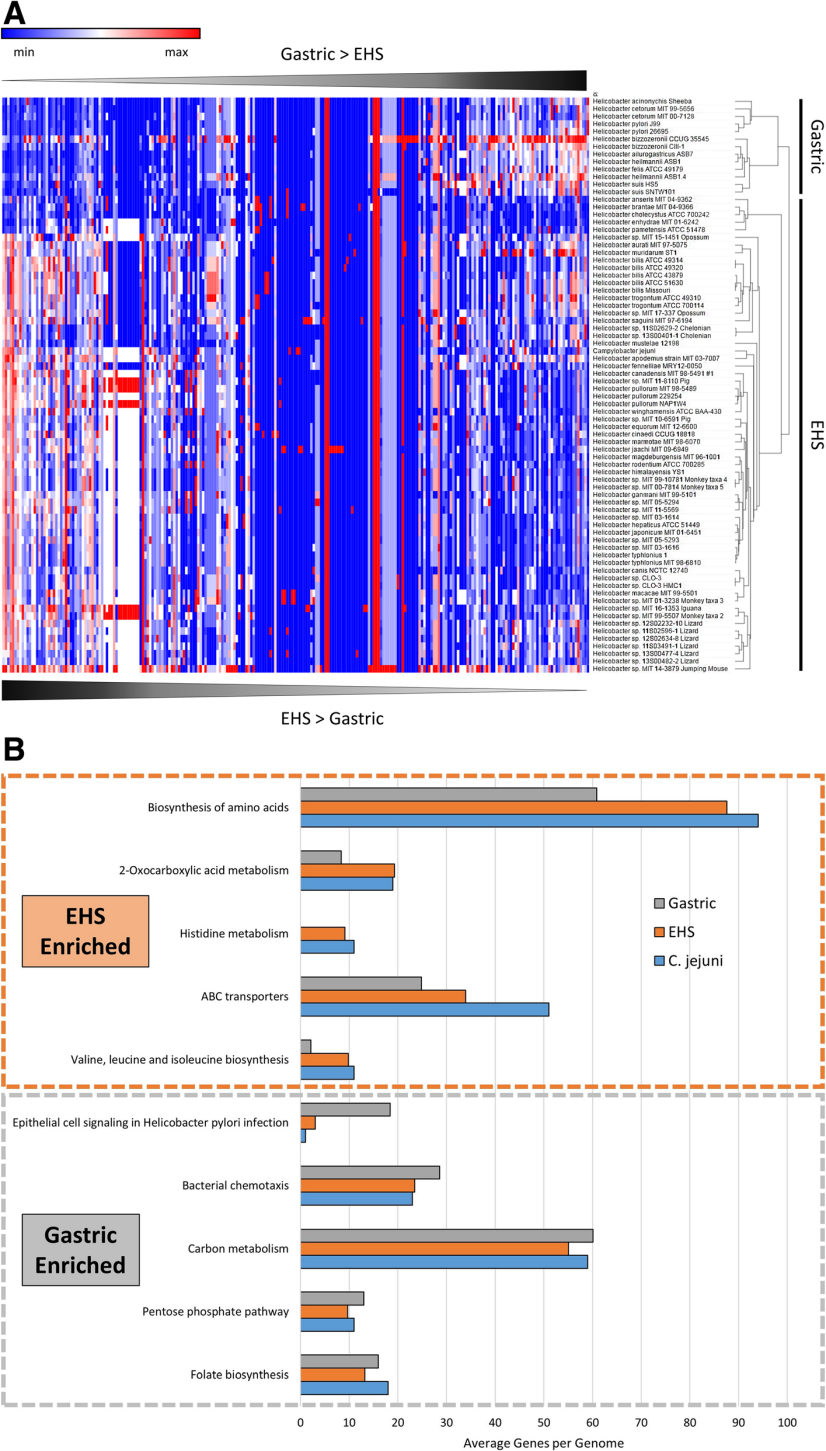
**a**) Heatmap and hierarchal clustering of KEGG metabolic and functional pathways according to relative gene abundance per genome. Gastric and EHS genomes clustered into distinct clades from each other, and enterohepatic genomes clustered more closely with *C. jejuni*. See supplement for table heatmap data (Additional file 1: Table S5). **b**) Top 10 enriched KEGG metabolic and functional pathways in gastric versus EHS genomes

To infer the potential physiological and pathogenic significance of the genetic heterogeneity between gastric species and EHS, genomes were analyzed by KAAS to assign protein coding sequences into functional classifications and metabolic pathways (i.e., KEGG pathways). Hierarchical clustering of KEGG pathway profiles organized genomes into gastric versus EHS designation, indicating specific metabolic functions/pathways differentiate these types (Fig. 3a, Additional file 1: Table S5). EHS genomes were enriched in genes functioning in de novo amino acid biosynthesis and metabolism of 2-oxocarboxylic acid (e.g., pyruvate and oxaloacetate) as well as ABC transporters (Fig. 3b). Gastric species genomes had more genes functioning in bacterial chemotaxis, carbon metabolism, the pentose phosphate pathway, and folate biosynthesis (Fig. 3b). Gastric species were also highly enriched for “Epithelial cell signaling in *Helicobacter pylori* infection,” which includes the virulence factors genes vacuolating cytotoxin and the cag type-IV secretion system (cag-T4SS); this is discussed in more detail later.

### Gastric and enterohepatic species have different metabolic potentials

**Fig. 4:**
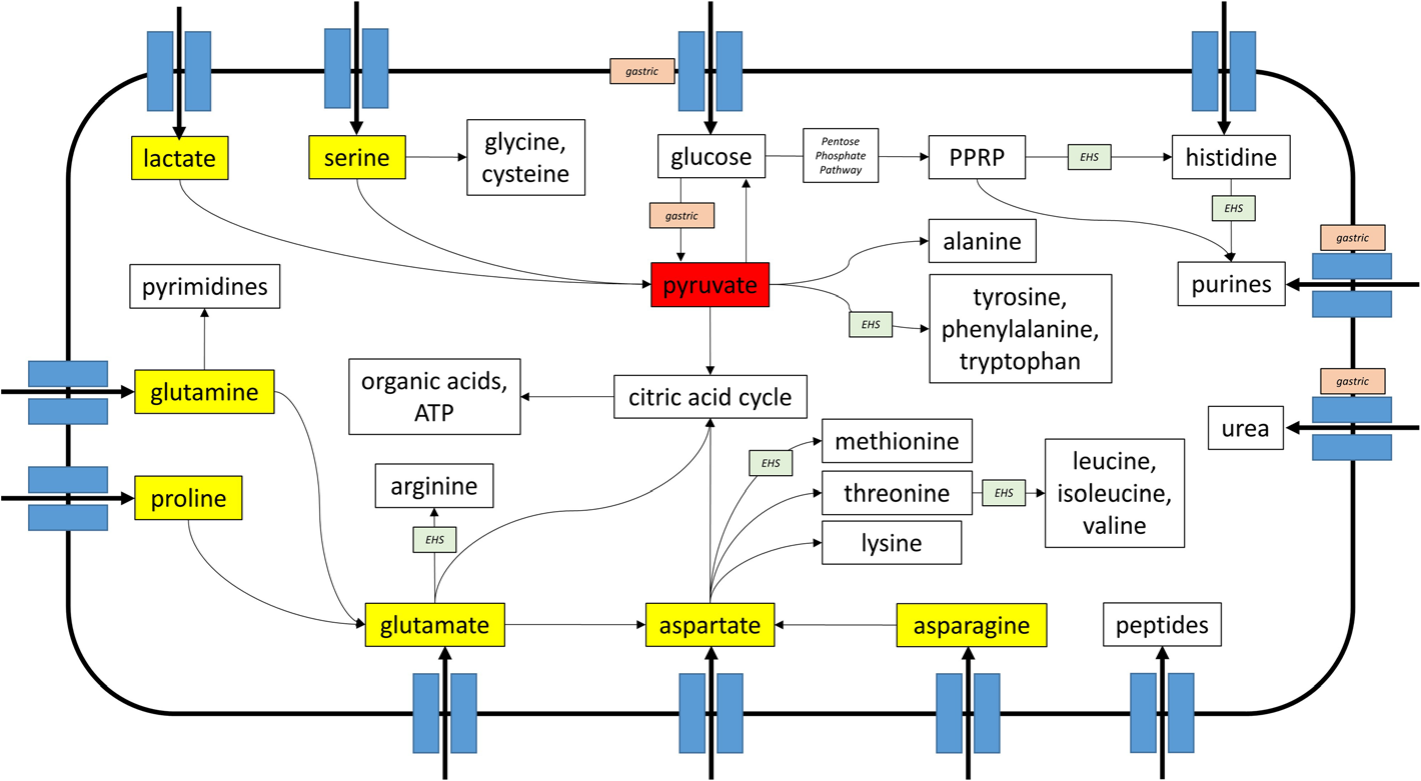
Graphic summary of major metabolic pathways in *Helicobacter spp*. Transporters and pathways enriched in/unique to gastric and EHS genomes are indicated. Also annotated are pyruvate (red box), the central metabolite in *Helicobacter spp*. metabolism, and other critical nutrients (yellow boxes) that are precursors for biosynthetic pathways and energy production. See Additional file 6: Figure S2, Additional file 3: Figure S3, Additional file 4: Figure S4 for expanded metabolic reconstruction diagrams

The above differences were further explored via reconstruction and comparison of metabolic pathways between 76 EHS and gastric genomes representative of all species and strains isolated from different host species (Additional file 1: Table S6). Metabolic reconstruction predicted that EHS cannot uptake or metabolize glucose or other simple sugars and instead are dependent on amino/organic acids to fuel metabolism. Gastric species are able to utilize glucose as well as amino/organic acids, but lack de novo biosynthetic pathways for several amino acids and purines commonly found in EHS. Gastric instead may rely on environmental uptake/salvage pathways to acquire these molecules. For all *Helicobacter spp.*, pyruvate appeared to be the central metabolite linking the networks for carbohydrates, amino acids, and nucleotides metabolism as well as the precursors for energy production (Fig. 4, Additional file 3: Figure S3, Additional file 4: Figure S4, Additional file 5: Figure S5). The differences predicted for carbohydrate, amino acid, and nucleotide metabolism between gastric and EHS genomes are discussed below as well as summarized in Fig. 4 and Tables 1, 2, 3.

**Table 1:** Uptake and Metabolism of Sugars and Organic Acids in Gastric versus EHS Genomes^a^

Metabolite	Uptake/Metabolism	*C. jejuni*	EHS 1	EHS 2	EHS 3	EHS 4	EHS 5	EHS 6	EHS 7	EHS 8	EHS 9	Gastric
Sugars	Glucose Transporter	0/1	1/12	0/11	0/2	0/3	0/11	0/5	0/6	0/4	1/8	10/13
	Glycolysis (Glucose → Pyruvate)	0/1	0/12	0/11	0/2	0/3	0/11	0/5	0/6	0/4	0/8	0/13
	Gluconeogenesis (Pyruvate → Glucose)	1/1	12/12	11/11	2/2	3/3	11/11	5/5	6/6	4/4	8/8	13/13
	Entner-Doudoroff Pathway (Glucose→ Pyruvate)	0/1	1/12	0/11	0/2	0/3	0/11	0/5	0/6	0/4	1/8	11/13
	Fucose → Pyruvate + Lactate	1/1	0/12	0/11	2/2	0/3	0/11	0/5	0/6	0/4	2/8	0/13
Organic Acids	Lactate → Pyruvate	1/1	11/12	11/11	2/2	3/3	11/11	5/5	6/6	4/4	8/8	13/13
	Propionate → Pyruvate	1/1	2/12	10/11	2/2	1/3	10/11	2/5	4/6	0/4	3/8	3/13
	Citrate Transporter	1/1	3/12	0/11	0/2	1/3	2/11	1/5	0/6	0/4	0/8	4/13
	Alpha-Ketoglutarate Transporter	1/1	2/12	10/11	2/2	0/3	2/11	1/5	2/6	0/4	6/8	12/13
	C4-Dicarboxylate Transporter (e.g., Succinate, Fumarate, Malate, Aspartate)	1/1	12/12	11/11	2/2	2/3	11/11	2/5	6/6	4/4	6/8	13/13

^a^Number of genomes positive for uptake/metabolism per clade

**Table 2:** Amino Acid Uptake, Biosynthesis, and Metabolism in Gastric versus EHS Genomes^a^

Amino Acid Family	Uptake/Metabolism	Amino Acid/Metabolite	*C. jejuni*	EHS 1	EHS 2	EHS 3	EHS 4	EHS 5	EHS 6	EHS 7	EHS 8	EHS 9	Gastric
Serine Family	Transporter	Serine	1/1	11/12	7/11	2/2	3/3	10/11	4/5	3/6	4/4	8/8	13/13
		Alanine, Glycine	1/1	5/12	0/11	0/2	1/3	7/11	4/5	2/6	4/4	8/8	13/13
		Cysteine	1/1	12/12	11/11	2/2	3/3	11/11	4/5	6/6	0/4	6/8	12/13
	Biosynthesis/ Metabolism	Serine → Pyruvate (*sdaA*)	1/1	9/12	10/11	2/2	3/3	5/11	5/5	6/6	4/4	8/8	13/13
		Pyruvate → Alanine	0/1	0/12	11/11	2/2	1/3	0/11	0/5	0/6	0/4	7/8	11/13
		Glycerate-3P → Serine	1/1	12/12	11/11	2/2	3/3	11/11	5/5	6/6	0/4	7/8	13/13
		Serine → Glycine	1/1	12/12	11/11	2/2	3/3	11/11	5/5	6/6	4/4	8/8	13/13
		Serine → Cysteine	1/1	12/12	11/11	2/2	3/3	11/11	5/5	6/6	0/4	5/8	12/13
Aromatic Family	Transporter	Tryptophan	0/1	10/12	0/11	0/2	0/3	0/11	0/5	0/6	0/4	0/8	0/13
		Histidine	1/1	12/12	11/11	0/2	1/3	3/11	0/5	3/6	4/4	8/8	13/13
	Biosynthesis/ Metabolism	Tryptophan	1/1	10/12	8/11	2/2	3/3	0/11	5/5	6/6	0/4	8/8	12/13
		Phenylalanine	1/1	12/12	11/11	2/2	3/3	11/11	5/5	6/6	1/4	8/8	1/13
		Tyrosine	1/1	12/12	11/11	2/2	3/3	11/11	5/5	6/6	0/4	7/8	5/13
		Histidine	1/1	12/12	11/11	2/2	3/3	11/11	5/5	6/6	0/4	8/8	0/13
Aspartate Family	Transporter	Asparagine/Aspartate	1/1	12/12	11/11	2/2	3/3	11/11	4/5	6/6	0/4	6/8	12/13
		Threonine	1/1	0/12	0/11	0/2	0/3	0/11	0/5	0/6	0/4	0/8	0/13
		Leucine, Isoleucine, Valine	1/1	12/12	0/11	0/2	0/3	0/11	0/5	0/6	0/4	0/8	0/13
		Methionine	1/1	12/12	11/11	2/2	3/3	11/11	5/5	6/6	2/4	8/8	5/13
		Lysine	0/1	0/12	0/11	2/2	0/3	0/11	0/5	0/6	0/4	5/8	0/13
	Biosynthesis/ Metabolism	Asparagine → Aspartate (*ansB*)	1/1	12/12	11/11	2/2	2/3	11/11	5/5	6/6	4/4	8/8	13/13
		Glutamate → Aspartate (*aspB*)	1/1	12/12	11/11	2/2	3/3	11/11	5/5	6/6	4/4	8/8	13/13
		Aspartate → Threonine	1/1	12/12	11/11	2/2	3/3	11/11	5/5	6/6	4/4	8/8	13/13
		Pyruvate + Threonine → Valine, Leucine, Isoleucine	1/1	12/12	11/11	2/2	3/3	11/11	5/5	6/6	0/4	7/8	0/13
		Methionine	1/1	12/12	9/11	2/2	3/3	11/11	5/5	6/6	0/4	5/8	0/13
		Aspartate → Lysine	1/1	12/12	11/11	2/2	3/3	11/11	5/5	6/6	4/4	8/8	13/13
Glutamate Family	Transporter	Proline	1/1	8/12	11/11	2/2	2/3	3/11	5/5	6/6	3/4	8/8	13/13
		Glutamine	1/1	12/12	11/11	2/2	3/3	11/11	4/5	6/6	0/4	6/8	12/13
		Glutamate	1/1	12/12	11/11	2/2	3/3	11/11	4/5	6/6	3/4	8/8	13/13
		Arginine	0/1	0/12	2/11	0/2	0/3	0/11	0/5	0/6	0/4	4/8	11/13
		GABA	0/1	0/12	10/11	0/2	0/3	0/11	0/5	0/6	0/4	0/8	2/13
		Urea	0/1	1/12	10/11	0/2	0/3	4/11	0/5	0/6	0/4	8/8	13/13
	Biosynthesis/ Metabolism	Proline ↔ Glutamate (*putA*)	1/1	4/12	11/11	2/2	1/3	2/11	0/5	3/6	2/4	7/8	13/13
		Glutamine → Glutamate (*ggt*)	0/1	1/12	11/11	2/2	1/3	0/11	0/5	1/6	0/4	7/8	13/13
		Glutamate → Arginine	0/1	11/12	3/11	2/2	3/3	11/11	5/5	6/6	4/4	6/8	0/13
		Glutamate → GABA → Succinate	0/1	0/12	0/11	0/2	0/3	0/11	0/5	0/6	0/4	0/8	6/13
		Arginine → Urea	0/1	5/12	9/11	0/2	1/3	8/11	2/5	4/6	0/4	6/8	11/13
		Urea → CO_2_ + 2 NH_3_(urease)	0/1	1/12	10/11	0/2	0/3	4/11	0/5	0/6	0/4	8/8	13/13

^a^Number of genomes positive for uptake/metabolism per clade

**Table 3:** *de novo* Biosynthesis and Salvage of Purines in Gastric versus EHS Genomes^a^

Uptake/Metabolism	*C. jejuni*	EHS 1	EHS 2	EHS 3	EHS 4	EHS 5	EHS 6	EHS 7	EHS 8	EHS 9	Gastric
Histidine Transporter	1/1	12/12	11/11	0/2	1/3	3/11	0/5	3/6	4/4	8/8	13/13
PRPP → Histidine	1/1	12/12	11/11	2/2	3/3	11/11	5/5	6/6	0/4	8/8	0/13
Purine Transport/Salvage	1/1	4/12	0/11	2/2	0/3	0/11	0/5	0/6	4/4	8/8	13/13
*de novo* Purine Biosynthesis	1/1	12/12	11/11	2/2	3/3	11/11	5/5	6/6	4/4	8/8	3/13

^a^Number of genomes positive for uptake/metabolism per clade

#### Carbohydrate metabolism

All gastric species (except *H. heilmannii* ASB1 and *H. suis*) and only two EHS (*H. mustelae* and *H. apodemus*) have glucose permease genes for uptake of environmental glucose (Table 1). Unlike other prototypical enteric bacterial species like *Escherichia coli*, all *Helicobacter spp.* do not have a functional glycolysis pathway to metabolize glucose into pyruvate because they lack phosphofructokinase (Additional file 1: Tables S2 and S7), which is a rate limiting step catalyzing the formation of fructose 1,6-bisphosphate from fructose-6-phosphate. Alternatively, these gastric species and select EHS likely metabolize glucose into pyruvate via the Entner-Doudoroff (ED) pathway (Table 1). Thus, EHS appear to be asaccharolytic like *C. jejuni*, which also lacks a glucose uptake and metabolism pathway [19, 20]. While *C. jejuni* is also considered to be asaccharolytic [19, 20], some rare strains harbor genetic loci to uptake and convert glucose into pyruvate via the ED pathway [21, 22] or uptake and metabolize the simple sugar fucose into pyruvate and lactaldehyde [23–25]. Lactaldehyde can be subsequently converted into pyruvate [23–25]. The alterative glucose metabolism locus was not identified in any EHS genomes, but fucose metabolism loci were identified in four EHS *(H. anseris* and the novel reptile isolates *Helicobacter sp.* 11S03491–1, *Helicobacter sp.* 11S02629–2, and *Helicobacter sp.* 13S00401–1) (Table 1), indicating select EHS may be able to utilize alternative carbohydrate sources.

All *Helicobacter spp.* require carbohydrates as critical intermediate metabolites and for macromolecules (e.g., LPS and glycosylated surface proteins). The carbohydrate precursors for these requirements likely arise via converting pyruvate into glucose via the gluconeogenesis pathway, which is complete in all *Helicobacter spp.* Consequently, the asaccharolytic nature of EHS suggests a reliance on organic and amino acids to fuel gluconeogenesis and satisfy their carbon, nitrogen, and energy demands.

#### Organic and amino acid metabolism

All gastric and EHS genomes have lactate permease and lactate dehydrogenase genes which enable the uptake and conversion of lactate into pyruvate (Table 1). Lactate can support *H. pylori* growth in vitro as the sole carbon source, and mutation of the L-lactate dehydrogenase gene both ablated this growth in vitro as well as in vivo stomach colonization [26, 27]. In the stomach and lower intestine, lactate can arise as a byproduct from host and microbial metabolism [28, 29]. Additionally, a majority of EHS and a few gastric species also encoded short chain fatty acid transporters and the enzymatic pathways to metabolize propionate to pyruvate and succinate (Table 1). In the lower intestine, propionate and similar short chain fatty acids are byproducts of microbial metabolism of indigestible dietary carbohydrates [29] and can be catabolized as a carbon/energy source for some bacteria like *Salmonella spp* [30, 31].

Serine, proline, glutamine, glutamate, asparagine, and aspartate appear to be important carbon and nitrogen sources for all *Helicobacter spp.* genomes because they can be directly imported and serve as precursors for de novo synthesis pathways (Fig. 4, Additional file 3: Figure S3, Additional file 4: Figure S4). Nearly all *Helicobacter spp.* encode genes for a serine transporter and *sdaA* allowing the transport and conversion of serine to pyruvate (Table 2 and Additional file 3: Figure S3, Additional file 4: Figure S4). In *C. jejuni*, disruption of serine transport or *sdaA* significantly impaired its growth in vitro and colonization of chickens [32]. This suggests that serine may also have a significant contribution in *Helicobacter spp.* metabolism, especially for EHS that appear to lack the metabolic mechanisms to produce pyruvate. Additionally, glutamate and aspartate can enter and fuel the citric acid cycle (Fig. 4, Additional file 6: Figure S2). Proline and glutamine can be converted to glutamate via the *putA* and *ggt* genes, respectively, in gastric species, but only in select EHS clades (Table 2). Nearly all EHS but no gastric genomes contain the de novo synthesis pathway to produce arginine from glutamate (Table 2). Conversely, arginine transport is almost exclusively encoded in gastric, but not EHS genomes (Table 2). Asparagine and glutamate can be metabolized to aspartate by the *aspB* and *ansB* genes in all almost all *Helicobacter spp.* genomes (Table 2). Aspartate is the precursor in the de novo biosynthesis pathways for methionine, lysine, threonine, and the branched amino acids valine, leucine, and isoleucine (Fig. 4, Additional file 4: Figure S4). Gastric genomes lack the pathways for methionine, valine, leucine, and isoleucine synthesis, while EHS in general encode these pathways (Table 2).

de novo synthesis pathways for serine, glycine, cysteine, tryptophan, tyrosine, lysine, and threonine appear to be present in most gastric and EHS (Table 2). Gastric species can synthesize alanine from pyruvate, while only select EHS clades have this capability (Table 2). Conversely, phenylalanine and histidine de novo synthesis are absent in gastric but present in EHS (Table 2). Interestingly, not all *Helicobacter spp.* genomes compensate for missing de novo amino acid synthesis pathways with specific transporters. For example, gastric genomes lack transporters and the de novo synthesis pathway for branched amino acids. Both gastric and EHS may overcome these deficiencies by importing and metabolizing small peptides supported by the presence of peptidases and transporters for this function (Additional file 1: Tables S2, S7).

#### Citric acid cycle

The citric acid cycle (CAC) metabolizes pyruvate to oxaloacetate to reduce NAD+ and FAD, which are later used to generate ATP via the electron transport chain. CAC intermediates are also extracted to serve as precursors for the de novo synthesis of several amino acids, nucleotides, and fatty acids (Additional file 3: Figure S3). The CAC for all *Helicobacter spp.*, except for EHS clades 2 and 3, are incomplete because the gene for succinyl-CoA synthetase is missing (Additional file 1: Tables S2, S7). Therefore, the CAC appears to divide into an oxidative (C6) branch from citrate to succinyl-CoA and a reductive (C4) branch from oxaloacetate to succinate. Similar to *C. jejuni*, only the EHS clades 2 and 3 have complete “aerobic-like” CAC because they encode succinyl-CoA synthetase. In general, almost all *Helicobacter spp.* genomes encode C4-dicarboxylate transporters that allow uptake and replenishing of succinate, fumarate, and malate into the CAC, while only select *Helicobacter spp.* genomes encode transporters for citrate and alpha-ketoglutarate (Table 1).

#### Nucleotide metabolism

Glutamine is the precursor for pyrimidine de novo biosynthesis, and along with glutamine transporters, gastric and EHS are capable of de novo pyrimidine synthesis (Fig. 4, Additional file 4: Figure S4, Additional file 5: Figure S5). Conversely, phosphoribosyl pyrophosphate (PRPP) and histidine are the precursors for de novo purine biosynthesis (Fig. 4, Additional file 6: Figure S2, Additional file 4: Figure S4). While all *Helicobacter spp.* genomes can synthesize PRPP from glucose via the non-oxidative branch of the pentose phosphate pathway, only EHS genomes encode the pathways to metabolize PRPP into histidine and then into purines (Table 3). Gastric species, as result of this deficiency, harbor a salvage pathway that includes transporters and enzymes for uptake and metabolic interconversion of purines (Table 3). Previous in silico predictions have noted that *H. pylori* cannot biosynthesize the purine precursor inositol monophosphate (IMP), and it has been experimentally shown that *H. pylori* growth in vitro is dependent on the salvage of purines from the environment [33]. Select EHS clades also harbor this purine salvaging mechanism.

Interestingly, EHS clade 8, which includes *H. brantae*, *H. cholecystus*, *H. pametensis*, and *H. enhydrae*, lacked many of the features conserved in most EHS genomes. For example, these genomes were missing 5/8 enzymes in the CAC (Additional file 1: Tables S2, S7). This is the only EHS clade to rely on purine salvage instead of de novo synthesis from histidine as well (Table 3). Other generally conserved amino acid transporters and metabolism pathways for cysteine, tryptophan, phenylalanine, tyrosine, methionine, valine, leucine, and isoleucine present in EHS were also not found in this clade (Table 2). EHS clade 8 seems to be an outlier that is likely compensated by novel genes and metabolic pathways not readily apparent in our analysis.

### Virulence factor profiles of enterohepatic species differ compared to gastric species

**Fig. 5:**
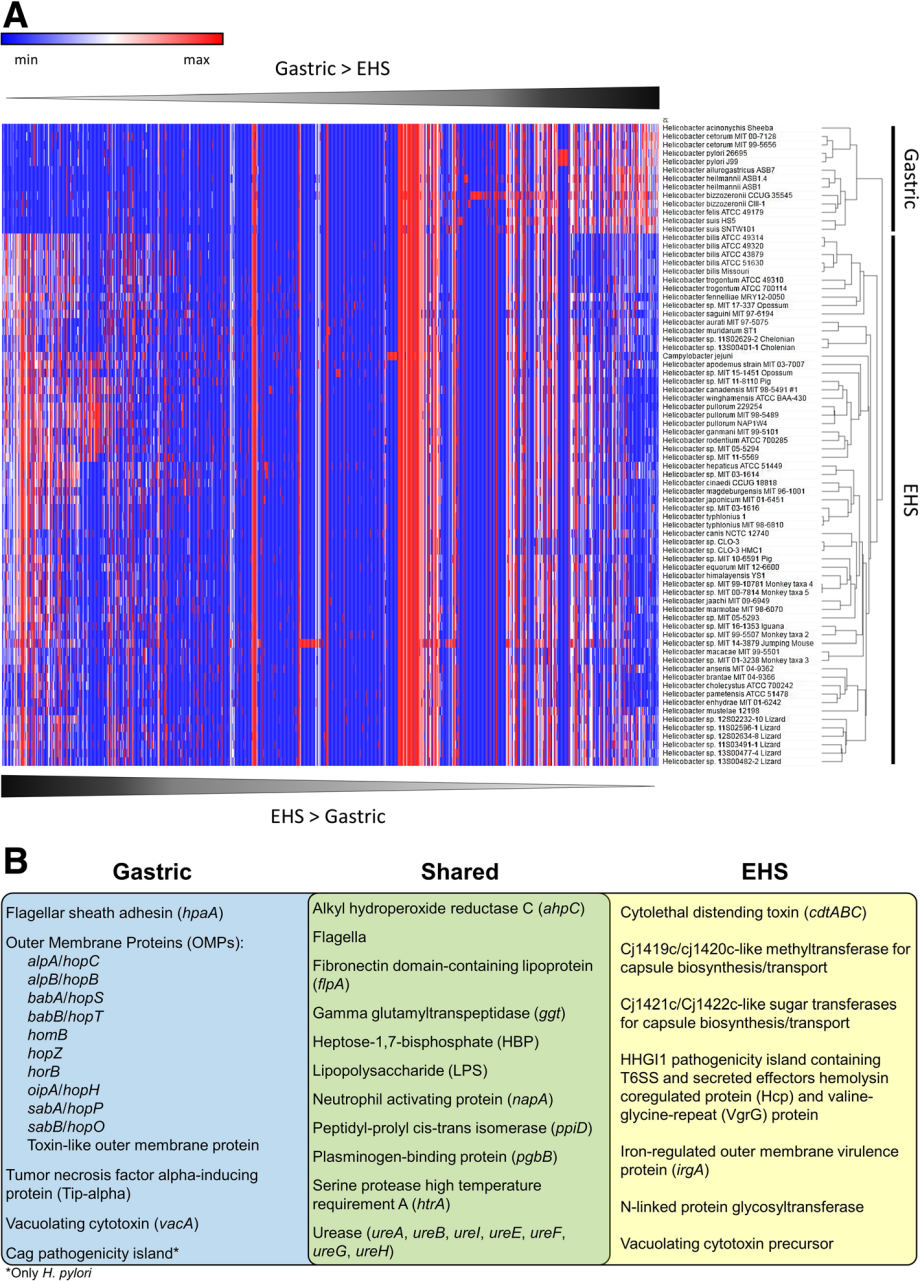
**a**) Heatmap and hierarchal clustering of virulence factor genes according to relative abundance per genome. Gastric and EHS genomes clustered into distinct clades from each other, and enterohepatic genomes clustered more closely with *C. jejuni*. See supplement for table heatmap data (Additional file 1: Table S9). **b**) Venn diagram of representative virulence factor genes shared and unique between gastric and EHS genomes

Both gastric species and EHS are frequently associated with gastrointestinal inflammation and cancer. While numerous pathogenic mechanisms have been identified and characterized in *H. pylori*, these are less understood for other gastric species and EHS. Additionally, it is largely unknown if gastric species versus EHS have conserved similarities and differences in virulence factor gene profiles. To identify and compare potential virulence factors profiles between gastric and EHS genomes, a BLASTP analysis was performed using the VICTORs and VFDB virulence factor databases and known *Helicobacter spp.* virulence factors genes described in the literature. Gastric genomes on average contained 492.4 ± 33.7 homologous virulence factor genes compared to 534.0 ± 44.9 genes in EHS genomes and 603 genes in *C. jejuni* (Additional file 1: Table S8). Numerous virulence factor homologs were shared in all *Helicobacter spp.* genomes; however, hierarchical clustering indicated gastric and EHS have different overall virulence factor profiles (Fig. 5a, Additional file 1: Table S9). Notable virulence factors shared and different between gastric and EHS genomes are summarized in Fig. 5b and discussed below. An expanded description of these genes can be found in the Additional file 7 results/discussion section.

#### Cytotoxins

*H. pylori* expresses vacuolating cytotoxin A (VacA), a pore-forming cytotoxin that produces vacuoles in gastric epithelial cells that results in apoptosis and can trigger inflammation events [34–36]. It has also been shown to inhibit T cell activation and proliferation allowing immune evasion and colonization persistence. *H. cetorum* and *H. acinonychis* were the only other gastric species beside *H. pylori* to encode *vacA* genes (Additional file 1: Table S8). Select EHS also encode homologs to vacuolating cytotoxin precursor annotations originally described in *H. canadensis* MIT 98–5491 [37] (Additional file 1: Table S8). It is unknown if any of these *vacA*-like genes in gastric and EHS have virulence properties. The only known cytotoxin for EHS is cytolethal distending toxin (CDT), a double-stranded DNA nuclease that has been shown experimentally to promote pro-inflammatory pathology and induce pro-carcinogenic DNA damage in the intestine by infection with *H. hepaticus* and other select EHS [38–41]. Genes for *cdt* were detected in 53/81 EHS genomes analyzed, but not in any gastric species (Additional file 1: Table S6).

#### Secretion systems

The cag pathogenicity island in *H. pylori* is composed of a type-IV secretion system (T4SS) that injects the cytotoxin-associated gene A (CagA) effector into host cells to exert a variety of cytotoxic effects on cell junction integrity, proliferation, morphology, and dysregulation of intracellular signaling cascades [35]. Patients with *H. pylori* strains harboring the cag pathogenicity island have a significantly greater risk of developing gastric cancer [35]. *H. pylori* strains also encode three other T4SS gene islands called ComB, Tfs3, and Tfs4 which function in DNA uptake/transfer from the environment and/or secretion of virulence factors that promote gastric inflammation [42–45]. A BLAST analysis identified that nearly all gastric and EHS genomes contained homologs for *virB*, *virD*, and other T4SS components organized in genetic loci (Additional file 1: Table S10). ComB, Tfs3, or Tfs4 system fragments with sequence identities > 80% were detected in the genomes of *H. cetorum*, *H. suis*, and *H. acinonychis* (Additional file 1: Table S10), which has also been previously described by Delahay and co-workers [46]. However, for most genomes, hits often had sequence identities < 50% to *cag*, *comB*, *tfs3*, or *tfs4* genes (Additional file 1: Tables S8 and S10), making it difficult to conclude whether these homologs clearly are representative of the T4SS found in *H. pylori*. As shown by Fischer and co-workers, T4SS genes from different systems in *H. pylori* genomes (e.g., Tfs3 vs. Tfs4) can have low to high sequence similarity, but should still be considered distinct from each other [47]. The loci identified in gastric and EHS genomes herein may encode novel T4SS that are assembled and function differently compared to those known for *H. pylori*. Whether these putative T4SS represent DNA-uptake systems or have virulence properties requires future experimental studies.

*H. hepaticus* harbors the HHGI1 pathogenicity island that contains a contiguous 11 gene cluster for type VI secretion system components (HH_0242-HH_0252) and includes the secreted effectors called hemolysin co-regulated protein (Hcp) and valine-glycine-repeat (VgrG) protein [10, 48, 49]. *H. hepaticus* strains lacking this loci induce less severe hepatic and lower intestinal inflammatory pathology [48–50]. *H. bilis*, *H. cinaedi*, *H. fennelliae*, *H. hepaticus*, *H. japonicum*, some strains of *H. pullorum*, *H. saguini*, *Helicobacter sp.* MIT 03–1614, *Helicobacter sp.* MIT 05–5294, *Helicobacter sp.* MIT 11–5569, *H. didelphidarum* MIT 17–337 Opossum, *Helicobacter sp.* MIT 14–3879 Jumping Mouse, and *H. trogontum*, but no gastric genomes, also appeared to harbor homologous loci for this type VI secretion system and effector proteins (Additional file 1: Table S11).

#### Membrane-associated factors

Gastric species were enriched in homologs for the *hor*, *hom*, and *hop* gene families of outer membrane proteins (OMPs) [51] (Additional file 1: Tables S8, S12). In *H. pylori* and other gastric species, these OMPs function as adhesins to the stomach mucosal and epithelial surface and are highly susceptible to mutations that facilitate rapid adaptation and colonization in the gastric environment. Homologs to the prototypical *H. pylori hor*, *hom*, and *hop* gene families of OMPs genes were only identified in 6 EHS genomes. *H. saguini* contained two hypothetical proteins with autotransporter beta-domains (InterProScan domain: IPR005546) and homology to the toxin-like outer membrane protein gene from *H. pylori* [52] (Additional file 1: Table S8, S12). *Helicobacter sp.* 13S00482–2 Lizard, *Helicobacter sp.* MIT 10–6591 Pig, *Helicobacter sp.* 12S02232–10 Lizard and encode *hopZ* homologs, while *horB* homologs were detected in *Helicobacter sp.* 12S02634–8 Lizard and *Helicobacter sp.* 13S00477–4 Lizard.

Aside from these OMPs, all EHS genomes (except *Helicobacter sp.* 11S02629–2) contained several genes homologous to virulent adhesins/OMPs, such as iron-regulated outer membrane virulence protein (*irgA*) from other enteric pathogens including *Campylobacter spp.*, *Salmonella enterica*, and *Escherichia coli* (Additional file 1: Table S8, S13). Fibronectin domain-containing lipoprotein (*flpA*) genes were conserved in all gastric and EHS genomes, indicating the potential to adhere to fibronectin in the extracellular matrix of the intestinal epithelium [53–55]. Furthermore, additional novel outer membrane proteins were identified in all gastric and EHS genomes by screening outer membrane/hypothetical annotations for outer membrane InterProScan domains (Additional file 1: Table S14). On average, outer membrane-associated virulence factor genes were twice as frequent in gastric species compared to the EHS and *C. jejuni* genomes (gastric: 15.5 ± 8.3; EHS: 8.2 ± 5.4; *C. jejuni*: 9 genes per genome).

All EHS genomes contained methyltransferases and sugar transferases genes not found in any gastric genomes. These genes are homologous to the *C. jejuni cj1419c*/*cj1420c* and *cj1421c*/*cj1422c* genes (Additional file 1: Table S8) that are putatively involved in the biosynthesis and transport of the extracellular capsule, which has been described to have virulence properties relating to resistance killing by complement proteins in vitro as well as the activation of pro-inflammatory cytokine expression and colonization persistence in vivo [56–58]. Also, EHS but not gastric genomes encoded an N-linked protein glycosyltransferase homologous to general protein glycosylation (*pgl*) genes found in *C. jejuni*. These glycosyltransferases mediate conjugation of simple sugar complexes to amino acids residues on extracellular proteins yielding glycans that enable adherence and invasion of host epithelial cells in vitro and in vivo [59]. Glycosylation may also protect extracellular proteins from degradation by host proteases, also promoting survival [60]. Previously, it has been reported that a functional N-Linked protein glycosylation system exists in the EHS *H. pullorum* [61]. Thus, EHS may have different mechanisms for forming and decorating their extracellular surfaces compared to gastric species.

#### Lipopolysaccharide

Lipopolysaccharide (LPS) is a component of the outer cell membrane that facilitates host colonization and modulates immune responses. Biosynthesis of the lipid A, core oligosaccharide, and O-antigen domains of LPS by *H. pylori* has only been partially elucidated, but in general follows that of other gram-negative bacteria [62]. Little is known about LPS biosynthesis and structure in other gastric species and EHS. The pathway for lipid A biosynthesis appears to be conserved among gastric and EHS genomes (Additional file 1: Table S15). Interestingly, all gastric and EHS are missing a homolog for *lpxM*, the last enzyme in lipid A biosynthesis pathway, while EHS clade 2 and 3 are also missing a homolog for *lpxL*, the penultimate enzyme. This suggests that alternative, unidentified genes compensate for *lpxM* and *lpxL* function in *Helicobacter spp.*

LPS by gram-negative pathogens are typically potent induces of immune responses; however, *H. pylori* constitutively modifies its lipid A to dramatically suppress LPS immune reactivity. Modification of lipid A for *H. pylori* includes removal of phosphates groups by *lpxE* and *lpxF*, addition of a phosphoethanolamine (*P*EtN) moiety by *eptA*, removal of keto-deoxyoctulosonate (Kdo) sugar residue by Kdo hydrolase, and removal of 3-*O*-linked acyl chains by *lpxR* [62]. All of these modification enzymes are also found in the *H. acinonychis* and *H. cetorum* strains, whereas other gastric and EHS genomes, except *Helicobacter sp.* MIT 10–6591 Pig, *Helicobacter sp.* MIT 16–1353 Iguana, *H. canadensis* MIT 98–5491, and *H. pullorum* NAP8W25, encode at least one homolog of these genes (Additional file 1: Table S15).

The core oligosaccharide is synthesized by a series of glycosyltransferases. Only a few glycosyltransferases have been defined in *H. pylori*, while the remaining are unidentified. Several glycosyltransferases are conserved in all gastric and EHS genomes, including the *rfac* and *rfaF* heptosyltransferase genes (Additional file 1: Table S15). However, InterProScan analysis found that gastric and EHS genomes are enriched in different profiles of novel glycosyltransferases genes that suggests the synthesis and structure of the core oligosaccharide differs among *Helicobacter spp.* (Additional file 1: Table S8). The O-antigen of *H. pylori* is composed of saccharides yielding Lewis antigens with structural similarity to host cell surface antigens. As a result, *H. pylori* can mimic host Lewis antigens thereby providing a mechanism of immune evasion or production of destructive auto-antibodies against the host [62]. Homologs for N-acetylglucosamine transferase (*rfaJ*), galactose transferase, and alpha-1,3-fucosyltransferase (*futA*), or alpha-1,2-fucosyltransferase (*futC*) responsible for the synthesis of the O-antigen were identified in all gastric and EHS genomes (Additional file 1: Table S15). All genomes from clade 1 (except *H. apodemus*), *H. cholecystus*, *H. macacae*, *Helicobacter sp.* 12S02634–8 Lizard, and *Helicobacter sp.* MIT 01–3238 Monkey taxa 3 lacked homologs for all of these genes (Additional file 1: Table S15). Novel glycosyltransferases identified in the Helicobacter genomes may also contribute to the biosynthesis and modification of the O-antigen as well.

## Discussion

In this whole-genus comparative study, we have identified genetic features that differentiate gastric and enterohepatic *Helicobacter spp.* on the basis of phylogenetics, genomic characteristics, metabolic pathways, and virulence factors genes. The results from these analyses provide new insight into the conserved and contrasting physiological and pathogenic mechanisms that have evolved in gastric species versus EHS to colonize and potentially cause pathology in their respective host intestinal environments.

We found that whole-genome phylogenetics more effectively organized Helicobacter genomes as belonging to either gastric or enterohepatic clades than the commonly used 16s rRNA sequence comparison. Our whole-genome phylogenetic tree resembled those constructed by Gilbert and co-workers [63] as well as by Smet and co-workers [17]. Unlike Gilbert and co-workers’ tree, we considered *H. mustelae* and the 6 novel lizards *Helicobacter spp.* isolates as EHS and not gastric species. Smet and co-workers also classified *H. mustelae* as an EHS, but did not include *H. anseris*, an EHS isolated from the feces of the geese [64], nor the 6 novel lizards *Helicobacter spp.* in their analysis. The 6 novel lizard *Helicobacter spp.* isolates were cultured from cloacal swabs [63], so it is unclear if these organisms genuinely colonize the stomach, lower intestinal tract, or both. While *H. mustelae* is known to colonize and cause gastric disease in ferrets, this organism is commonly detected in the feces of juvenile ferrets, suggesting it could transit out of the stomach and potentially colonize the lower intestinal tract [15, 65]. Furthermore, phylogenetic organization by 16s and 23s rRNA gene similarity has found that *H. mustelae* more closely clusters with EHS than gastric species [11]. The lipid profile of *H. mustelae* is also enriched in hexadecanoic fatty acids, which has been described to be a characteristic of EHS and rare for gastric *Helicobacter spp.* [66]. Concordant with these results, we found that *H. mustelae* shared substantially more genes with EHS than gastric genomes, although genes unique to *H. mustelae* not found in other EHS, like *ureA2B2*, may contribute to its prominent gastric colonization.

According to our whole-genome phylogenetic tree, EHS could be divided into nine different clades. Individual clades did not cluster EHS according to host species, suggesting that evolution for the lower intestinal tract may have occurred independent of host species. Likewise, the orthogroups different between EHS clades mainly comprised hypothetical proteins, which obfuscates interpretation of what genetic factors delineates different EHS clades. Nevertheless, the whole-genome phylogenetic tree and drastically contrasting repertoire of orthogroups between gastric and EHS genomes indicates that a core set of genetic characteristics have evolved to dictate whether a *Helicobacter spp.* will colonize and cause pathology at the stomach or lower intestinal tract. Species like *H. mustelae* may have a blend of these genetic determinants to allow colonization at both sites. Therefore, we performed metabolic reconstruction and analysis for virulence factor genes to understand the potential physiological and pathogenic mechanisms that differentiate gastric and enterohepatic species.

Our metabolic reconstruction predicted that gastric and enterohepatic species have fundamentally different requirements for carbohydrate, amino acid, and nucleotide substrates to fuel metabolism. Compared to their gastric counterparts, EHS appear to have restricted sources for carbohydrates and nitrogen sources. Most striking is the inability of EHS to utilize simple sugars like glucose and subsequent reliance on amino and organic acids to fuel metabolism. Glutamine/glutamate, asparagine/aspartate, serine, and proline appear to be the most critical amino acids that fuel EHS metabolism because they can be readily acquired from the environment and enter metabolic pathways rapidly as pyruvate or directly into the CAC. As a result, the metabolic triangle of pyruvate-phosphoenolpyruvate-oxaloacetate connecting the CAC with gluconeogenesis (see Additional file 6: Figure S2, Additional file 3: Figure S3) appear particularly important in the metabolism of *Helicobacter spp.*, especially EHS because it is the only mechanism for producing carbohydrates from amino and organic acids via gluconeogenesis. Additionally, EHS are enriched in de novo biosynthesis pathways for several amino acids as well as purines that are absent in gastric species. The predicted inability of gastric species to synthesize the amino acids histidine, leucine, methionine, phenylalanine, and valine agrees with experimental evidence showing *H. pylori* requires media supplemented with these amino acids for in vitro grow in the absence of serum [67]. Unexpectedly, transporters are not always present in gastric and EHS to compensate for de novo biosynthetic deficiencies, suggesting novel transporters and/or metabolic pathways may be present in their respective genomes.

Evolutionary adaptation for survival in the stomach versus the large intestine may have influenced the predicted metabolic differences we detected between gastric and EHS genomes. This includes the drastically different anatomy, physiology, and possibly tissue-centric microbiomes of the gastric compartment versus the intestinal tract. The stomach and proximal small intestine are more acidic than the lower intestine and are the primary sites for nutrient digestion and absorption [68]. In the lower intestine, indigestible carbohydrates and proteins predominate and are primarily processed by the resident microbiota [68]. Bacterial species colonizing the stomach and small intestine (e.g., *H. pylori* and *Lactobacillus reuteri*, respectively) often have smaller size genomes with a restricted number of biosynthetic pathway genes because they occupy a nutrient rich environments [69]. Conversely, we observed that EHS, like other bacterial species that colonize the lower intestine, typically have larger size genomes and more diverse biosynthetic pathway genes, likely enabling them to adapt and utilize a variety of available nutrients [69]. Consequently, the larger genomes of EHS may endow them with increased metabolic flexibility compared to gastric species because EHS must be more resourceful to survive.

From our analysis, we observed that gastric species are enriched in methyl-accepting chemotaxis proteins and two-component signaling proteins compared to EHS. An abundance of these chemotaxis genes may give gastric species an advantage within the stomach to respond to environment stimuli with flagellated movement away from acidic pH levels and towards necessary nutrient gradients close to gastric epithelia. EHS, despite similar mucous-colonizing ability, likely have less access to basic nutrient molecules (e.g., simple sugar and free amino acids) than gastric species and therefore may have acquired de novo biosynthetic pathways, such as amino acids, to enable survival.

Microbiota differences in the stomach versus lower intestinal tract should also be considered as candidates that have influenced Helicobacter evolution. Aside from gastric *Helicobacter spp.*, the stomach does not have a complex microbiota unlike the lower bowel, which suggests reduced competition for colonization niches and nutrients. In comparison, the lower intestine is abundantly colonized by diversity of bacteria, fungi, and viruses, all of which may be competing for the colonization niches and nutrients needed by EHS for survival. However, our metabolic analysis also found that microbiome-derived nutrients like lactate and propionate may also benefit EHS and suggests that cooperative relationships could be essential for EHS colonization and pathogenicity within the intestinal tract. One study found that germfree mice infected with *H. hepaticus* only develop significant typhlocolitis if co-infected with *L. reuteri* [70]. Another study observed that *H. hepaticus*-induced intestinal pathology is exacerbated or attenuated depending on the composition of the murine microbiome [71]. It would be informative to further study how interactions with intestinal microorganisms influence the physiology and pathogenic potential of EHS.

It is important to appreciate that many EHS colonize not only the large intestine (e.g., cecum and colon), but have been detected in the gall bladder, biliary tract, and liver as well. In order to survive in this diversity of intestinal and extraintestinal niches, EHS may have experienced an evolutionary pressure to acquire/maintain a repertoire of flexible metabolic pathways. This may have been unnecessary for gastric species given their restricted niche. For some strains of *C. jejuni*, it has been shown that tropism and successful colonization of the intestinal tract versus the liver is dictated by γ-glutamyltranspeptidase (GGT) and a secreted isoform of asparaginase (AnsB), respectively [72].

GGT is an outer membrane-associated enzyme that metabolizes host glutamine into glutamate and ammonia, which are then imported by the bacterial cell as precursors to fuel metabolic needs. *ggt* expression is necessary for colonization persistence and the development of inflammation-induced pathology by *H. pylori* [73], *H. suis* [74], and *C. jejuni* [75]. We detected *ggt* homologs in all gastric species as well as select EHS genomes (see Table 2, Additional file 1: Table S6). Previously, it has been reported that *H. bilis* (EHS clade 2) contains two different *ggt* gene annotations: one that is enzymatically active and a second that has mutations in conserved functional regions rendering it inactive [76]. Interestingly, all EHS genomes from clade 2 also contain two different *ggt* gene annotations for the active gene and its putatively inactive paralog. GGT activity by *Helicobacter* and *Campylobacter spp.* has also been shown to yield an anti-proliferative effect to rapidly dividing intestinal epithelial and immune cells due to depletion of host glutamine reserves [76–80]. Therefore, *ggt* genes may be important factor that promotes intestinal tissue tropism and pathogenic potential in certain EHS.

AnsB catalyzes the conversation of asparagine to aspartate. While nearly all *Helicobacter spp.* have a homolog for *ansB*, only select gastric and EHS genomes encoded an isoform with predicted signal-peptide required for secretion (Additional file 1: Table S6). In *C. jejuni*, secreted AnsB, but the not cytoplasmic isoform, was required for significant liver tissue tropism [72]. EHS often isolated from the liver, like *H. hepaticus* and *H. bilis*, were found to encode the secreted *ansB* gene. Based on our predictions from the metabolic reconstruction, glutamine and asparagine are key precursors of carbon and nitrogen for EHS, and acquisition of these nutrients by GGT and AnsB may facilitate their enterohepatic tropism.

Urease plays an essential role in stomach colonization by metabolizing urea into ammonia in order neutralize stomach acid needed to permit survival in the gastric compartment. Isogenic mutants of gastric species like *H. pylori* [81] or *H. mustelae* [82] lacking urease cannot establish persistent stomach colonization. As expected, urea uptake and urease genes were identified in all gastric genomes. Interestingly, species from several EHS clades (see Table 2) also harbored the urease genetic loci. Unexpectedly, *H. enhydrae*, the novel species isolated from the stomachs of southern sea otters, lacks urease genes and activity [16]. Urease activity by these select EHS may facilitate survival in the acidic gastrointestinal environment during transmission to new hosts. However, urease may also produce ammonia for nitrogen assimilation rather than acid neutralization in the intestine (pH ~ 6.1) and liver (pH ~ 7.4) [83]. Experimentally, it has been shown that urease deficiency in isogenic *H. hepaticus* mutants does not impair cecal colonization, but does prevent hepatic colonization and consequently attenuates hepatic pathology compared to the wild-type strain [83]. Additionally, urease-expressing EHS (e.g., *H. hepaticus* and *H. bilis*) but not urease-negative species (e.g., *H. cinaedi* or *H. rodentium*) are capable of inducing cholesterol gallstones and associated hepatobiliary inflammatory pathology in mice [84]. Likewise, only urease-expressing EHS can precipitate calcium in vitro [85]. Thus, urease expression by EHS may promote hepatobiliary colonization and pathology.

Aside from metabolic differences, we found that gastric *Helicobacter spp*. and EHS encode a wide diversity of virulence genes ranging from adhesins, cytotoxins, and survival factors. Some virulence genes are shared among all *Helicobacter spp.*, while other contribute to the unique virulence profiles that differentiate gastric and EHS from each other. Thus, fundamental differences appear to exist in the mechanisms by which gastric and EHS are capable of eliciting pathogenic infection in their hosts. For example, while flagella subunits and assembly genes were conserved in all *Helicobacter spp.* genomes, gastric genomes encode flagellar sheath adhesin (*hpaA*), a protein that protects flagellin subunits from depolymerization in low pH environments [86]. The presence of *hpaA* in gastric but not EHS genomes indicates that virulence factor genes have also undergone evolutionary pressures to fit their colonization niche. Previously, it has been reported that O-antigen structure for *H. pylori* also varies depending on the pH of in vitro culture [62]. Therefore, the pH difference in the colonization niche preference of gastric versus EHS may also affect their O-antigen structure. In agreement with our genetic analysis suggesting differences in LPS biosynthesis, phenotypic characterizations of LPS have found substantial structural and immune-reactive heterogeneity among gastric species and EHS [87] and emphasize the need to further experimentally validate the pathogenic significance of LPS in *Helicobacter spp.*

Interestingly, some virulence genes identified also have overlapping metabolic functions. For example, GGT is important for glutamate acquisition and colonization, while simultaneously yielding deleterious effects to the host. These genes have been coined “nutritional virulence factors” in *C. jejuni* and other pathogens [88, 89] and may be an important new source for understanding the pathogenic determinants in *Helicobacter spp.* Identifying and characterizing if metabolically-associated genes have virulence properties would not only enhance understanding of how *Helicobacter spp.* maintain colonization in their different sites, but may also elucidate mechanisms utilized by these organisms to induce inflammatory pathology and cancers.

Several proteomics studies have indicated that gastric and enterohepatic *Helicobacter spp.* express different protein profiles. Fowsantear and co-workers showed there are significantly different proteomic profiles between several representative gastric and enterohepatic *Helicobacter spp.* [90]. Interestingly, these authors noted that *H. felis*, a gastric species isolated from felines, grouped more closely with other EHS, and *H. mustelae* did not cluster with *H. pylori* or other EHS. Another study by Kornilovs’ka and co-workers analyzing surface protein profiles of *H. pylori* and representative EHS found distinct differences among the *Helicobacter spp.* [91]. Antisera collected from rabbits immunized with sonicate from these *Helicobacter spp.* identified several surface proteins capable of inducing immunogenic host responses. A comparison of OMPs between *H. bilis* strains isolated from mice, dogs, rats, and gerbils to *H. pylori* found *H. bilis* strains have similar OMP profiles among each other, but were different compared to *H. pylori* [92]. These OMPs were also capable of inducing immunogenic host responses. Lastly, Hynes and co-workers showed that the proteomic profiles of *H. pylori* and several representative EHS were not only distinct but also changed differently among species when challenged by bile stress in vitro [93, 94]. A limitation of the aforementioned studies is that whole-genome sequences were not available for all species/species at the time of their analysis, thereby impairing cross-validation of differentially expressed proteins using mass spectrometry-based protein identification methods. In our study, while genomic annotation do not unequivocally confirm differences in biological function between gastric and enterohepatic *Helicobacter spp.*, the findings and datasets we present will facilitate further studies (such as transcriptomics, proteomics, metabolomics, and other phenotypic experiments) needed to validate our predictions regarding *Helicobacter spp.* physiology and pathogenic mechanisms.

## Conclusion

This genus-wide comparative analysis determined that gastric *Helicobacter spp.* and EHS can be differentiated on the basis distinct genetic features. Phylogenetic classification by whole-genome and ANI was found to be a more effective way to taxonomically identify and classify gastric and EHS and was more accurate than traditional 16s rRNA gene sequences. This is important because it provided a means by which the novel EHS included in this study could be differentiated as novel species or new strains of existing species. Furthermore, metabolic reconstruction revealed key differences in the uptake, biosynthesis, and metabolism of carbohydrates, amino acids, and nucleotides between gastric and EHS. These findings have enhanced our insights into how these organisms may have evolved and adapted to colonize their respective niches. Lastly, gastric species and EHS have overlapping as well as distinct virulence factor profiles. In addition to the canonical factors, and novel virulence gene homologs were identified in both gastric and EHS, thereby increasing the repertoire of possible virulence mechanisms. Most importantly, the findings from this study provide new opportunities in the future to experimentally probe how these metabolic and virulence genes affect colonization and pathogenicity of gastric species and EHS using in vitro and in vivo models.

## Methods

### Genome sequencing, assembly, and gene annotation

*Helicobacter spp.* were grown on trypticase soy agar plate with 5% sheep blood (Remel Laboratories, Lenexa, KS). The plates were incubated at 37 °C under microaerobic conditions in a vented jar containing N_2_, H_2_, and CO_2_ (80:10:10) for 48 h. Bacteria pellets were collected for isolation of genomic DNA using the MasterPure Complete DNA and RNA Purification Kit (Epicentre, Madison, WI) following the manufacturer’s protocol for bacterial cell samples. DNA libraries were prepared by the Sequencing Core at the Forsyth Institute (Cambridge, MA) using NextraXT for sequencing of 2 × 150 or 2 × 250 paired-end reads by Illumina MiSeq. Raw sequence reads were decontaminated of adapter sequences and quality trimmed to a Phred quality score (Q) ≥ 10 using BBDuk from the BBMap package version 36.99 (http://sourceforge.net/projects/bbmap/). Decontaminated reads were then de novo assembled into contigs with SPAdes hosted by the PATRIC server (Pathosystems Resource Integration Center) [95, 96].

Several genomes previously sequenced by our lab [97–100] were re-assembled by first performing decontamination and quality trimming on raw sequencing reads using BBDuk followed by de novo contigs assembly with SPAdes, as described above. All publically available gastric (only four representative *H. pylori* genomes) and EHS genomes were downloaded from the National Center for Biotechnology Information database (NCBI) (on 9/1/2017). In total 110 genomes were included in the analysis, representing 17 gastric speceis and 45 EHS (Additional file 1: Table S1). The genome for *Campylobacter jejuni* subsp. jejuni NCTC 11168 = ATCC 700819 was also downloaded and included in the analysis as a closely related outgroup. All genomes were annotated with RAST on the PATRIC server for consistency in subsequent analyses.

### Bioinformatic analyses

16s rRNA phylogenetic trees were constructed using MegAlign from the Lasergene software package (DNAStar Inc., Madison, WI) by aligning full length 16s rRNA gene sequences with ClustalW followed by phylogenetic tree construction using the neighbor-joining method. The Bacterial Pan Genome Analysis (BPGA) tool was used to identify orthologous gene clusters with USEARCH at 50% identify threshold for subsequent pan-genome phylogenetic tree making by the neighbor-joining method [101]. OrthoANI-usearch was used to calculate average nucleotide identity (ANI) between genomes in order to differentiate species at a 95% similarity threshold [102]. Orthofinder was used to identify orthologous genes and assign them into orthogroups [103]. KAAS (KEGG Automatic Annotation Server) was used to assign KO (KEGG Orthology) annotations for metabolic reconstruction and functional predictions (parameters: program: GHOSTX; method: SBH; GENES data set: hsa, dme, ath, sce, pfa, eco, sty, hin, pae, nme, hpy, rpr, mlo, bsu, sau, lla, spn, cac, mge, mtu, ctr, bbu, syn, aae, mja, afu, pho, ape, hpj, hhe, hac, hms, cje, wsu) [104]. BLAST2GO [105] and InterProScan 5 [106] were used to further predict and validate the domains and functions of protein gene annotations. Virulence factor genes were identified by BLASTP analysis of genomes against the VICTORs [107] and VFDB [108] virulence factor databases as well as against known *Helicobacter spp.* virulence factors genes described in the literature. BLASTP parameters were set at sequence identity ≥25%, sequence coverage ≥75%, and E-value ≤10e-10.

Data was organized and analyzed using Python 2.7.14 (https://www.python.org/) and Pandas v0.22.0 (https://pandas.pydata.org/). Heatmaps and hierarchical clustering of data was performed using Morpheus (Broad Institute, Cambridge, MA; https://software.broadinstitute.org/morpheus/). Phylogenetic trees and dendrograms were created with FigTree v1.4.3 (http://tree.bio.ed.ac.uk/software/figtree/).

## Additional files

Additional file 1: **Table S1.** Average Nucleotide Identity. **Table S2**. Genome Annotations. **Table S3**. Gastric and EHS Clade Orthogroups. **Table S4.** *H. mustelae* Orthogroups. **Table S5**. KEGG Heatmap. **Table S6**. Genome Metadata and Characteristics. **Table S7**. Metabolism Pathways per Genome. **Table S8**. Virulence Factor BLAST Results. **Table S9.** Virulence Factor Heatmap. **Table S10**. *H. pylori* T4SS. Table S11 *H. hepaticus* T6SS. **Table S12**. *H. pylori* Outer Membrane Proteins. **Table S13**. Other Outer Membrane Proteins. **Table S14**. Novel Membrane-Associated Proteins. **Table S15**. LPS Biosynthesis. (XLSX 147858 kb)

Additional file 2: Supplementary Results/Discussion Section (Additional file 7: Figure S1). (DOCX 47 kb)

Additional file 3: **Figure S3**. Expanded diagram of carbohydrate, amino acids, and nucleotide metabolic pathways reconstructed in Helicobacter genomes. Nutrients that can be imported from the environment to fuel metabolism are labeled with blue boxes. The metabolic triangle between phosphoenolpyruvate (PEP), pyruvate, and oxaloacetate (red boxes) links the Entner-Doudoroff (ED) pathway and gluconeogenesis with the citric acid cycle (CAC; contained within pink box). Selected enzymes are indicated in circles with solid arrows showing their reactions. Dashed arrows indicate multi-enzyme reactions to different biosynthetic pathways (green boxes). Abbreviations: 4-aminobutyrate-2-oxoglutarate transaminase (puuE), acetate kinase (ackA), acetyl-coenzyme A synthetase (Acs), aldehyde dehydrogenase A (Ald), asparaginase (AnsB), aspartase (AspA), aspartate aminotransferase (AspB), Entner-Doudoroff (ED) pathway, gamma-aminobutyric acid (GABA), gamma-glutamyltranspeptidase (GGT), glutamate decarboxylase (GAD), lactate dehydrogenase (LDH), non-oxidative pentose phosphate pathway (PPP), phosphoribosyl pyrophosphate (PRPP), phosphotransacetylase (pta), proline dehydrogenase (PutA), serine dehydratase (SdaA), succinate-semialdehyde dehydrogenase (gabD), phosphoenolpyruvate (PEP). (JPG 383 kb)

Additional file 4: **Figure S4**. Expanded diagram of amino acids biosynthesis pathways reconstructed in Helicobacter genomes. Enzymes are labeled in boxes with their enzyme code (E.C.) or gene abbreviations and solid arrows showing their reactions. Dashed arrows indicate multi-enzyme reactions to different biosynthetic pathways. Abbreviations: alanine (Ala), arginine (Arg), asparagine (Asn), aspartic acid (Asp), cysteine (Cys), glutamic acid (Glu), glutamine (Gln), glycine (Gly), histidine (His), isoleucine (Ile), leucine (Leu), lysine (Lys), methionine (Met), phenylalanine (Phe), proline (Pro), serine (Ser), threonine (Thr), tryptophan (Trp), tyrosine (Tyr), valine (Val), non-oxidative pentose phosphate pathway (PPP), asparaginase (AnsB), aspartate aminotransferase (AspB), gamma-glutamyltranspeptidase (GGT), proline dehydrogenase (PutA), serine dehydratase (SdaA), 5-Aminoimidazole-4-carboxamide ribonucleotide (AICAR), phosphoribosyl pyrophosphate (PRPP), erythrose 4-phosphate (E4P), glycerate-3P (3PG), phosphoenolpyruvate (PEP), pyruvate (Pyr), oxaloacetate (Oxa). (JPG 334 kb)

Additional file 5: **Figure S5**. Expanded diagram of purine (left) and pyrimidine (right) nucleotide biosynthesis pathways reconstructed in Helicobacter genomes. Enzymes are labeled in boxes with their enzyme code (E.C.) and solid arrows showing their reactions. Dashed arrows indicate multi-enzyme reactions to different biosynthetic pathways. Abbreviations: phosphoenolpyruvate (PEP), pyruvate (Pyr), glucose-6-phosphate (G6P), phosphoribosyl pyrophosphate (PRPP), glutamine (Gln), uridine triphosphate (UTP), cytidine triphosphate (CTP), inosine monophosphate (IMP) adenine monophosphate (AMP), xanthine monophosphate (XMP), guanine monophosphate (GMP), guanine (Gua), xanthine (Xan), hypoxanthine (Hyx), adenine (Ade). (JPG 148 kb)

Additional file 6: **Figure S2**. Plot of genome sizes (average ± standard deviation) for EHS, gastric, and *C. jejuni* genomes. Red dashed line indicates average gastric genome size for comparison. (JPG 305 kb)

Additional file 7: **Figure S1.** A) Genome sizes were plotted against the number of annotated protein coding sequences (CDS) and GC content. For all Helicobacter genomes, a linear relationship existed for genome size versus number of annotated protein CDS (R^2^ = 0.8939). Three EHS genomes (*H. muridarum* ST1, *H. pametensis* ATCC 51478, and *H. cholecystus* ATCC 700242) and one gastric genome (*H. bizzozeronii* CCUG 35545) appeared to be outliers and are indicated in the graph. Diamonds (◆), gastric genomes; circles (●) EHS genomes; cross (**X**), *C. jejuni* genome. B) Genome sizes were plotted against the additive size of all protein CDS, RNA genes, and non-coding gene sequences. Linear relationships existed for genome size versus size of protein CDS (R^2^ = 0.9782) and non-coding gene sequences (R^2^ = 0.8057), but not for RNA gene sequences (R^2^ = 0.0076). (ZIP 458 kb)
