# Supplementary material for: Comparative genomics analysis to differentiate metabolic and virulence gene potential in gastric versus enterohepatic Helicobacter species
Source: BMC Genomics. 2018 Nov 20;19:830. doi: 10.1186/s12864-018-5171-2 (PMC6247508; doi:10.1186/s12864-018-5171-2)
Supplement: Supplementary file 2 — Supplementary Results/Discussion Section (Additional file 7: Figure S1). (DOCX 47 kb) [file 12864_2018_5171_MOESM2_ESM.docx]

**Comparative Genomics Analysis to Differentiate Metabolic and Virulence Gene Potential in Gastric Versus Enterohepatic *Helicobacter* Species**

Anthony Mannion (manniona@mit.edu), Zeli Shen (zshen@mit.edu), James G. Fox (jgfox@mit.edu)

Division of Comparative Medicine, Massachusetts Institute of Technology, Cambridge, MA, United States

***Supplemental Results/Discussion***

***Gross Genome Characteristics***

All EHS genomes except from clade 8 were on average larger in size compared to gastric genomes and *C. jejuni* (EHS: 1.98 ± 0.31 Mbp; gastric: 1.66 ± 0.10 Mbp; *C. jejuni*: 1.64 Mbp) (supplemental figure 2, supplemental table 6). Also, EHS genomes were annotated with more protein coding sequences (CDS) (2,083.59 ± 355.94) compared to gastric genomes (1,793 ± 186.44) and *C. jejuni* (1,688) (supplemental table 1). Gastric genomes had a higher average GC content compared to EHS genomes (42.68 ± 4.33 versus 36.25 ± 3.32) (supplemental table 6). When genome size and number of protein CDS for all species was plotted against each other, a linear relationship was observed, indicating that in general, larger *Helicobacter spp.* genomes encode more putative genes (supplemental figure 1A). Likewise, the total length of all protein CDS was directly proportional to the size of the genome (supplemental figure 1B). Interestingly, the total length of all non-coding sequences (i.e., a sequence not annotated as protein or RNA genes) was also directly proportional to the genome (supplemental figure 1B). The significance of this finding is unclear, but may suggest that these species have an increased tendency to uptake and integrate extracellular DNA into their chromosomes, which has been described in other bacterial species as a potential mechanism for environmental adaptation[[1](#_ENREF_1)]. In general, EHS are physically larger than gastric species, and this size difference may accommodate larger genomes and more gene products.

***Virulence Factors***

A number of virulence factors genes were shared or not between gastric and EHS genomes. Notable virulence factors genes the discussed below.

All gastric species but no EHS genomes encoded one or more genes for tumor necrosis factor alpha-inducing protein (*Tip-alpha*) (supplemental table 8), a secreted protein that has been found in *H. pylori* to enter and translocate into the nucleus of gastric epithelial cells to promote NF-κB-mediated expression of TNF‐α and other pro-inflammatory genes[[2](#_ENREF_2)]. Additionally, *Tip-alpha* expression levels by *H. pylori* were found to be significantly higher in patients with gastric cancer, suggesting it may directly promote pro-carcinogenic changes[[2](#_ENREF_2)]. Tip-alpha may also promote *H. pylori* survival and fitness as knockout strains of this gene had impaired colonization in the mouse stomach[[3](#_ENREF_3)].

AhpC detoxifies organic peroxides formed by reactive oxygen species produced by activated macrophages and other innate immune cells. The detoxification of these harmful molecules protects the bacteria from oxidative protein, lipid, and DNA damage, therefore enabling survival. An *ahpC* isogenic knockout of the EHS *H. cinaedi* was more sensitive to organic hydroperoxide toxicity and death *in vitro* and had impaired colonization in the cecum of mice[[4](#_ENREF_4)]. *H. pylori* mutants of *ahpC* failed to colonize the stomach of mice[[5](#_ENREF_5)]. Homologs for *ahpC* were identified in all *Helicobacter spp.* genomes, and therefore represents a conserved mechanism to protect against oxidative stress. However, the functions of AhpC in gastric versus EHS may be different. *H. pylori* *ahpC* mutants were more susceptible to hydrogen peroxide toxicity than its wild-type counterpart, while counterintuitively, *H. cinaedi* *ahpC* mutants more resistant to hydrogen peroxide toxicity than the wild-type strain. *H. cinaedi* *ahpC* mutants were found to compensate for loss of AhpC by enhancing catalase enzyme activity. This finding has also been reported for *H. hepaticus*[[6](#_ENREF_6)] and *C. jejuni*[[7](#_ENREF_7)]. In contrast, *H. pylori* requires AhpC to prevent inactivation of catalase by organic peroxides and cannot compensate for loss of AhpC by increasing or protecting catalase activity by other mechanisms[[8](#_ENREF_8)].

Neutrophil-activating protein (*napA*) is a chemoattractant and activator for neutrophils to produce reactive oxygen species and other pro-inflammatory mediators[[9](#_ENREF_9), [10](#_ENREF_10)]. Simultaneously, NapA has also been shown to protect *H. pylori* from oxidative stress possibly via ferroxidase activity, and its expression is compensatory upregulated in *ahpC* mutants[[9](#_ENREF_9), [10](#_ENREF_10)]. *H. pylori* isogenic mutants for *napA* have impaired colonization in the mouse stomach[[11](#_ENREF_11)]. Therefore, *napA* expression could serve as a general mechanism for *Helicobacter spp.* to promote inflammation, tissue damage, and survival from oxidative stressors during infection.

Peptidyl-prolyl cis-trans isomerases (PpiD) are enzymes that catalyze the transition from cis to trans bond conformers for proline residues in proteins, therefore facilitating more favorable thermodynamic states for protein folding and denaturation[[12](#_ENREF_12)]. In *H. pylori*, PpiD is a secreted factor frequently detected antigen in human patient gastroduodenal ulcers[[13](#_ENREF_13)]. Additionally, PpiD from *H. pylori* has been reported to induce apoptotic cell death in gastric epithelial cells *in vitro* by directly binding toll-like receptor 4 (TLR4) and activating apoptosis signal-regulating kinase 1(ASK1) signaling pathways[[14](#_ENREF_14)]. The detection of *ppiD* genes in all *Helicobacter spp.* genomes suggests a conserved immuno-stimulant antigen with potential pro-apoptotic functions toward stomach and lower intestinal epithelial cell barriers.

PgbB binds host plasminogen, which then can be activated into the serine protease plasmin or activate host procollegnases to degrade blot clot and extracellular matrix molecules such as fibrin, fibronectin, and collagen[[15](#_ENREF_15)]. In general, PgbB activity by *Helicobacter spp.* may promote the degradation of these matrices and potentially lead to significant tissue damage that later facilitates invasive and persistent infection within the stomach and lower intestines. *pgbB* genes were identified in all *Helicobacter spp.* genomes.

HtrA activity by *H. pylori* and *C. jejuni* has been implicated in E-cadherin cleavage which deteriorates junctional connections between intestinal epithelial cells and may impair barrier integrity[[16](#_ENREF_16)]. All *Helicobacter spp.* genomes encode *htrA* homologs. In *H. pylori*, the *HtrA* gene if deleted prevents growth, indicating it is essential for viability[[17](#_ENREF_17)]. Conversely, *C. jejuni* isogenic knockout mutants have been successfully created and have been used to show that while the *htrA* gene is not required for intestinal colonization in chicken or mice models, its expression promotes more severe inflammation and pathology in the intestine[[18-20](#_ENREF_18)]. It would be interesting to determine if the *htrA* gene is required for viability in other gastric and EHS and its virulence properties *in vitro* and *in vivo*.

Heptose-1,7-bisphosphate (HBP), an intermediate in the core oligosaccharide biosynthesis, has been shown to be an intracellular inducer of pro-inflammatory cytokine expression by host cells[[21](#_ENREF_21)]. All *Helicobacter spp.* encode homologs for *hldE*, the gene necessary to synthesize HBP from sugar precursors in the pentose phosphate pathway (supplemental table 15). While *H. pylori* can directly inject HBP into host cells via its Cag-T4SS, HBP is also secreted by other bacteria species to induce immune responses after endocytosis by the host cell[[22](#_ENREF_22)]. Other gastric and EHS do not encode Cag-T4SS, but their T4SS-like genes may enable HBP secretion and trigger host inflammation.

**References**

1. Blokesch, M., *Natural competence for transformation.* Curr Biol, 2016. **26**(23): p. 3255.

2. Suganuma, M., et al., *TNF-alpha-inducing protein, a carcinogenic factor secreted from H. pylori, enters gastric cancer cells.* Int J Cancer, 2008. **123**(1): p. 117-22.

3. Godlewska, R., et al., *Tip-alpha (hp0596 gene product) is a highly immunogenic Helicobacter pylori protein involved in colonization of mouse gastric mucosa.* Curr Microbiol, 2008. **56**(3): p. 279-86.

4. Charoenlap, N., et al., *Alkyl hydroperoxide reductase is required for Helicobacter cinaedi intestinal colonization and survival under oxidative stress in BALB/c and BALB/c interleukin-10-/- mice.* Infect Immun, 2012. **80**(3): p. 921-8.

5. Olczak, A.A., et al., *Association of Helicobacter pylori antioxidant activities with host colonization proficiency.* Infect Immun, 2003. **71**(1): p. 580-3.

6. Hong, Y., G. Wang, and R.J. Maier, *A Helicobacter hepaticus catalase mutant is hypersensitive to oxidative stress and suffers increased DNA damage.* J Med Microbiol, 2007. **56**(Pt 4): p. 557-62.

7. Palyada, K., et al., *Characterization of the oxidative stress stimulon and PerR regulon of Campylobacter jejuni.* BMC Genomics, 2009. **10**: p. 481.

8. Wang, G., et al., *Role of a bacterial organic hydroperoxide detoxification system in preventing catalase inactivation.* J Biol Chem, 2004. **279**(50): p. 51908-14.

9. Choli-Papadopoulou, T., et al., *Helicobacter pylori neutrophil activating protein as target for new drugs against H. pylori inflammation.* World J Gastroenterol, 2011. **17**(21): p. 2585-91.

10. Fu, H.W., *Helicobacter pylori neutrophil-activating protein: from molecular pathogenesis to clinical applications.* World J Gastroenterol, 2014. **20**(18): p. 5294-301.

11. Wang, G., et al., *Dual Roles of Helicobacter pylori NapA in inducing and combating oxidative stress.* Infect Immun, 2006. **74**(12): p. 6839-46.

12. Unal, C.M. and M. Steinert, *Microbial peptidyl-prolyl cis/trans isomerases (PPIases): virulence factors and potential alternative drug targets.* Microbiol Mol Biol Rev, 2014. **78**(3): p. 544-71.

13. Atanassov, C., et al., *Novel antigens of Helicobacter pylori correspond to ulcer-related antibody pattern of sera from infected patients.* J Clin Microbiol, 2002. **40**(2): p. 547-52.

14. Basak, C., et al., *The secreted peptidyl prolyl cis,trans-isomerase HP0175 of Helicobacter pylori induces apoptosis of gastric epithelial cells in a TLR4- and apoptosis signal-regulating kinase 1-dependent manner.* J Immunol, 2005. **174**(9): p. 5672-80.

15. Jonsson, K., et al., *Molecular cloning and characterization of two Helicobacter pylori genes coding for plasminogen-binding proteins.* Proc Natl Acad Sci U S A, 2004. **101**(7): p. 1852-7.

16. Wessler, S., G. Schneider, and S. Backert, *Bacterial serine protease HtrA as a promising new target for antimicrobial therapy?* Cell Commun Signal, 2017. **15**(1): p. 4.

17. Harrer, A., et al., *Overexpression of serine protease HtrA enhances disruption of adherens junctions, paracellular transmigration and type IV secretion of CagA by Helicobacter pylori.* Gut Pathog, 2017. **9**: p. 40.

18. Heimesaat, M.M., et al., *The role of serine protease HtrA in acute ulcerative enterocolitis and extra-intestinal immune responses during Campylobacter jejuni infection of gnotobiotic IL-10 deficient mice.* Front Cell Infect Microbiol, 2014. **4**: p. 77.

19. Heimesaat, M.M., et al., *The impact of serine protease HtrA in apoptosis, intestinal immune responses and extra-intestinal histopathology during Campylobacter jejuni infection of infant mice.* Gut Pathog, 2014. **6**: p. 16.

20. de Boer, P., et al., *Generation of Campylobacter jejuni genetic diversity in vivo.* Mol Microbiol, 2002. **44**(2): p. 351-9.

21. Stein, S.C., et al., *Helicobacter pylori modulates host cell responses by CagT4SS-dependent translocation of an intermediate metabolite of LPS inner core heptose biosynthesis.* PLoS Pathog, 2017. **13**(7): p. e1006514.

22. Pachathundikandi, K. and S. Backert, *Heptose 1,7-Bisphosphate Directed TIFA Oligomerization: A Novel PAMP-Recognizing Signaling Platform in the Control of Bacterial Infections.* Gastroenterology, 2018. **154**(4): p. 778-783.
