# Supplementary figures and images for: Comparative genomics analysis to differentiate metabolic and virulence gene potential in gastric versus enterohepatic Helicobacter species
Source: BMC Genomics. 2018 Nov 20;19:830. doi: 10.1186/s12864-018-5171-2 (PMC6247508; doi:10.1186/s12864-018-5171-2)

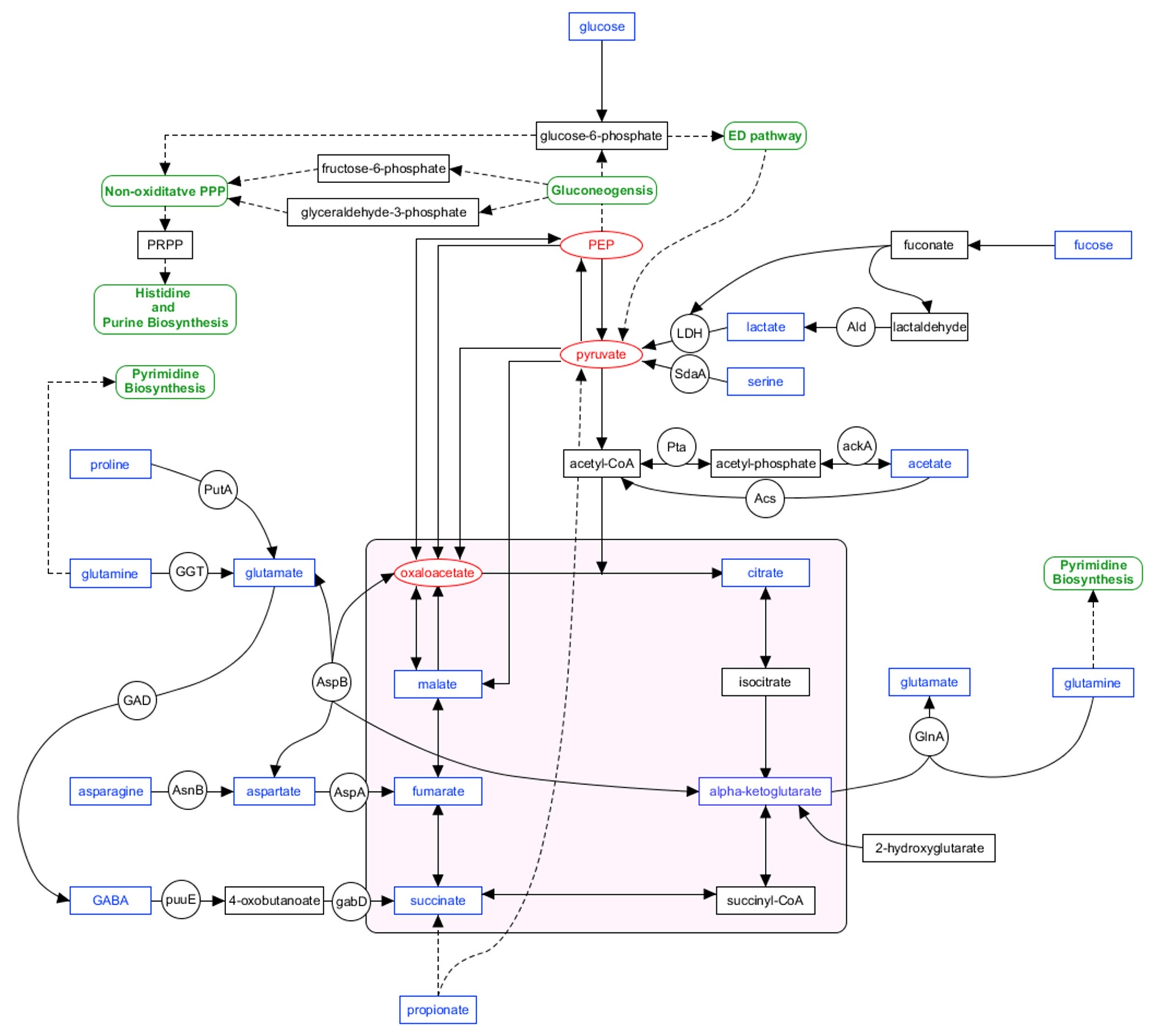

Supplement: Supplementary file 3 — Figure S3. Expanded diagram of carbohydrate, amino acids, and nucleotide metabolic pathways reconstructed in Helicobacter genomes. Nutrients that can be imported from the environment to fuel metabolism are labeled with blue boxes. The metabolic triangle between phosphoenolpyruvate (PEP), pyruvate, and oxaloacetate (red boxes) links the Entner-Doudoroff (ED) pathway and gluconeogenesis with the citric acid cycle (CAC; contained within pink box). Selected enzymes are indicated in circles with solid arrows showing their reactions. Dashed arrows indicate multi-enzyme reactions to different biosynthetic pathways (green boxes). Abbreviations: 4-aminobutyrate-2-oxoglutarate transaminase (puuE), acetate kinase (ackA), acetyl-coenzyme A synthetase (Acs), aldehyde dehydrogenase A (Ald), asparaginase (AnsB), aspartase (AspA), aspartate aminotransferase (AspB), Entner-Doudoroff (ED) pathway, gamma-aminobutyric acid (GABA), gamma-glutamyltranspeptidase (GGT), glutamate decarboxylase (GAD), lactate dehydrogenase (LDH), non-oxidative pentose phosphate pathway (PPP), phosphoribosyl pyrophosphate (PRPP), phosphotransacetylase (pta), proline dehydrogenase (PutA), serine dehydratase (SdaA), succinate-semialdehyde dehydrogenase (gabD), phosphoenolpyruvate (PEP). (JPG 383 kb) [file 12864_2018_5171_MOESM3_ESM.jpg]

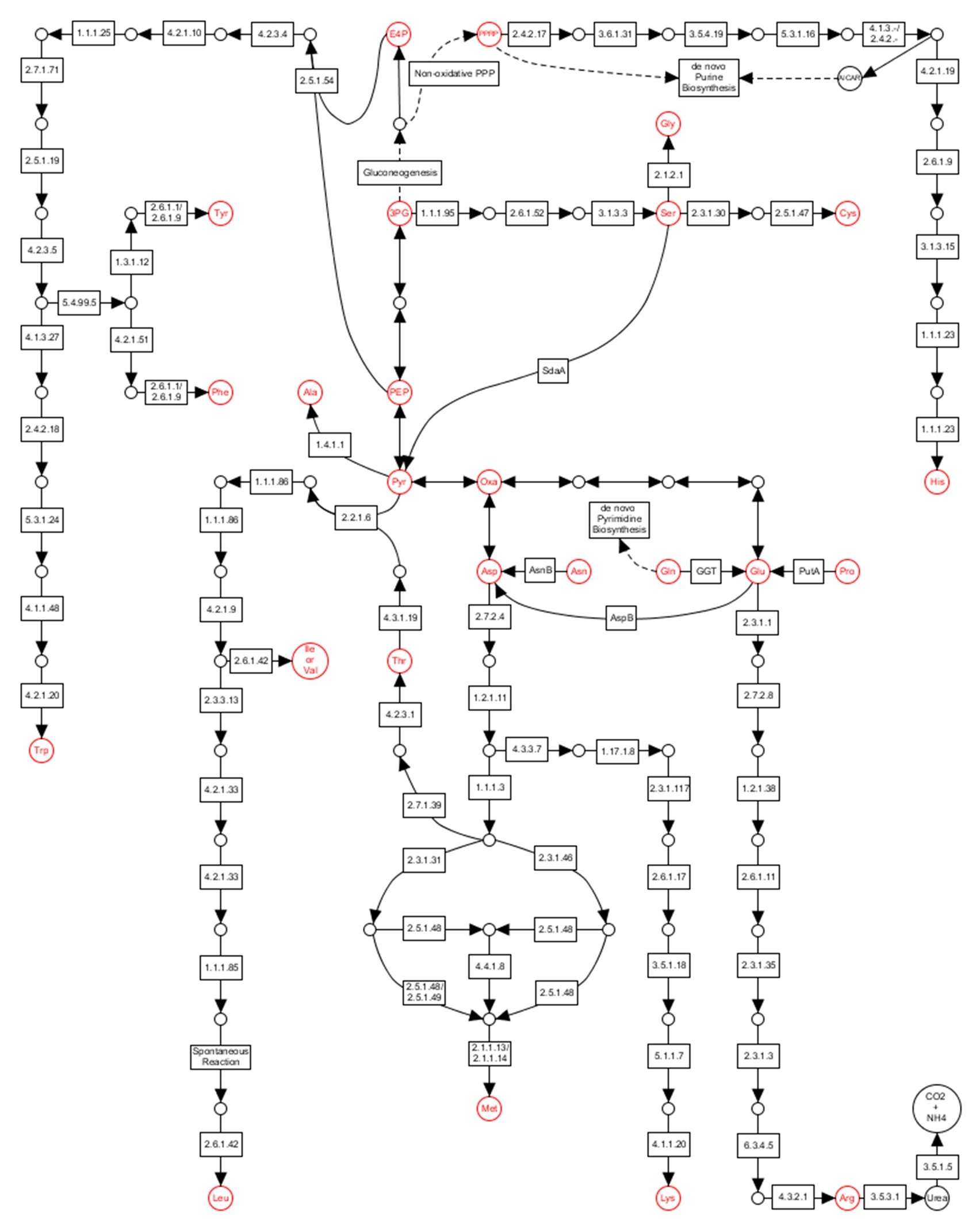

Supplement: Supplementary file 4 — Figure S4. Expanded diagram of amino acids biosynthesis pathways reconstructed in Helicobacter genomes. Enzymes are labeled in boxes with their enzyme code (E.C.) or gene abbreviations and solid arrows showing their reactions. Dashed arrows indicate multi-enzyme reactions to different biosynthetic pathways. Abbreviations: alanine (Ala), arginine (Arg), asparagine (Asn), aspartic acid (Asp), cysteine (Cys), glutamic acid (Glu), glutamine (Gln), glycine (Gly), histidine (His), isoleucine (Ile), leucine (Leu), lysine (Lys), methionine (Met), phenylalanine (Phe), proline (Pro), serine (Ser), threonine (Thr), tryptophan (Trp), tyrosine (Tyr), valine (Val), non-oxidative pentose phosphate pathway (PPP), asparaginase (AnsB), aspartate aminotransferase (AspB), gamma-glutamyltranspeptidase (GGT), proline dehydrogenase (PutA), serine dehydratase (SdaA), 5-Aminoimidazole-4-carboxamide ribonucleotide (AICAR), phosphoribosyl pyrophosphate (PRPP), erythrose 4-phosphate (E4P), glycerate-3P (3PG), phosphoenolpyruvate (PEP), pyruvate (Pyr), oxaloacetate (Oxa). (JPG 334 kb) [file 12864_2018_5171_MOESM4_ESM.jpg]

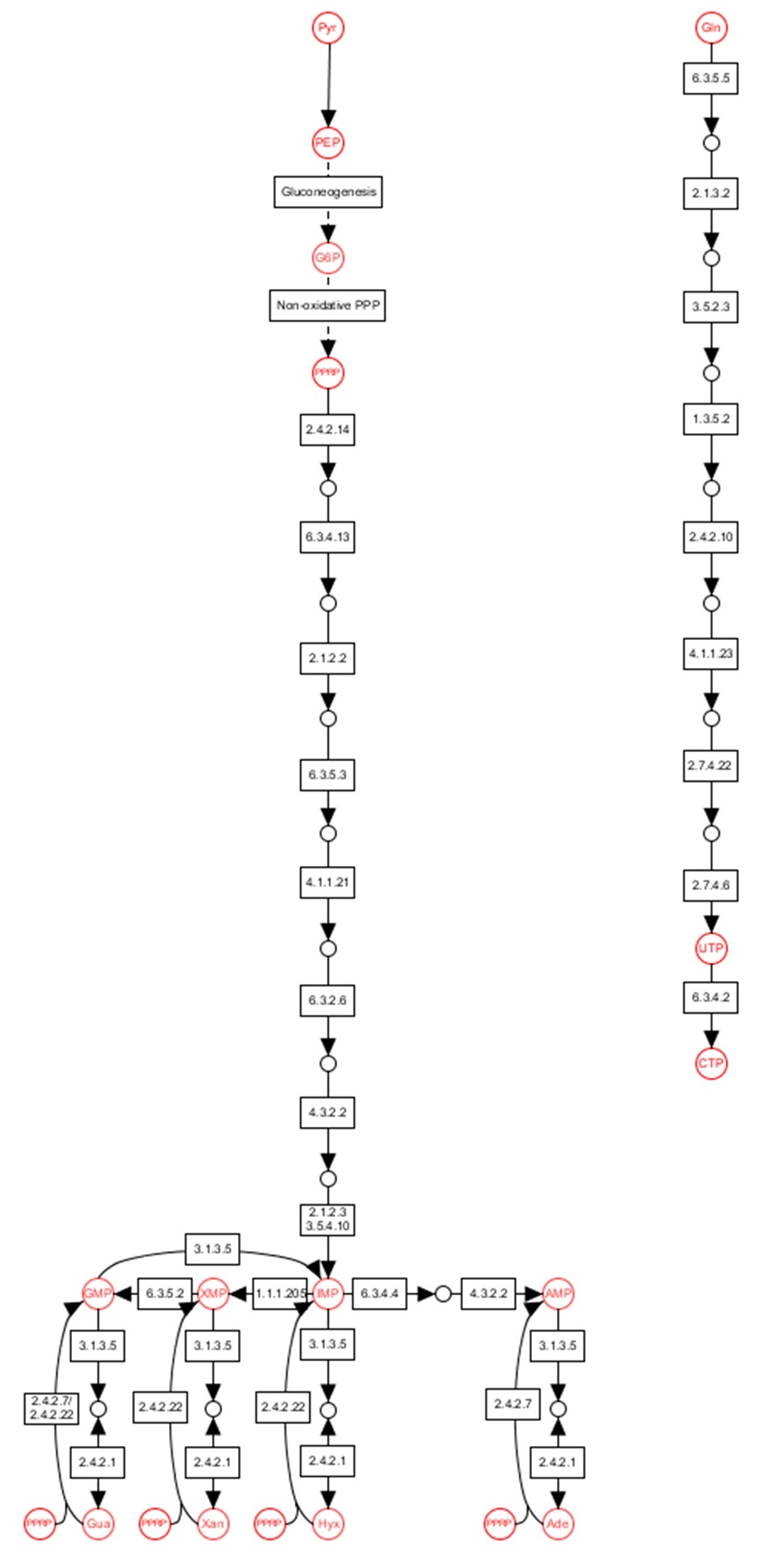

Supplement: Supplementary file 5 — Figure S5. Expanded diagram of purine (left) and pyrimidine (right) nucleotide biosynthesis pathways reconstructed in Helicobacter genomes. Enzymes are labeled in boxes with their enzyme code (E.C.) and solid arrows showing their reactions. Dashed arrows indicate multi-enzyme reactions to different biosynthetic pathways. Abbreviations: phosphoenolpyruvate (PEP), pyruvate (Pyr), glucose-6-phosphate (G6P), phosphoribosyl pyrophosphate (PRPP), glutamine (Gln), uridine triphosphate (UTP), cytidine triphosphate (CTP), inosine monophosphate (IMP) adenine monophosphate (AMP), xanthine monophosphate (XMP), guanine monophosphate (GMP), guanine (Gua), xanthine (Xan), hypoxanthine (Hyx), adenine (Ade). (JPG 148 kb) [file 12864_2018_5171_MOESM5_ESM.jpg]

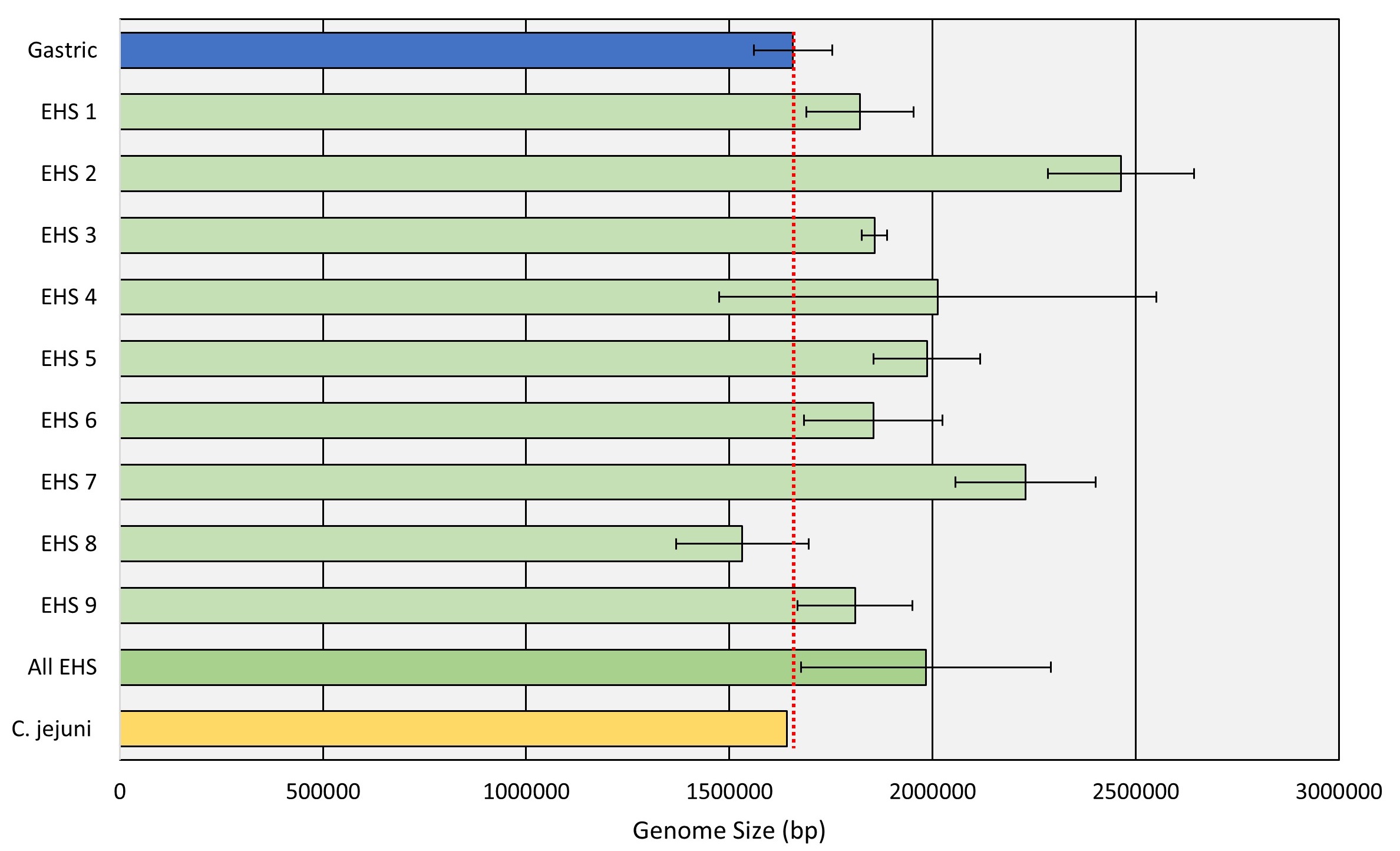

Supplement: Supplementary file 6 — Figure S2. Plot of genome sizes (average ± standard deviation) for EHS, gastric, and C. jejuni genomes. Red dashed line indicates average gastric genome size for comparison. (JPG 305 kb) [file 12864_2018_5171_MOESM6_ESM.jpg]

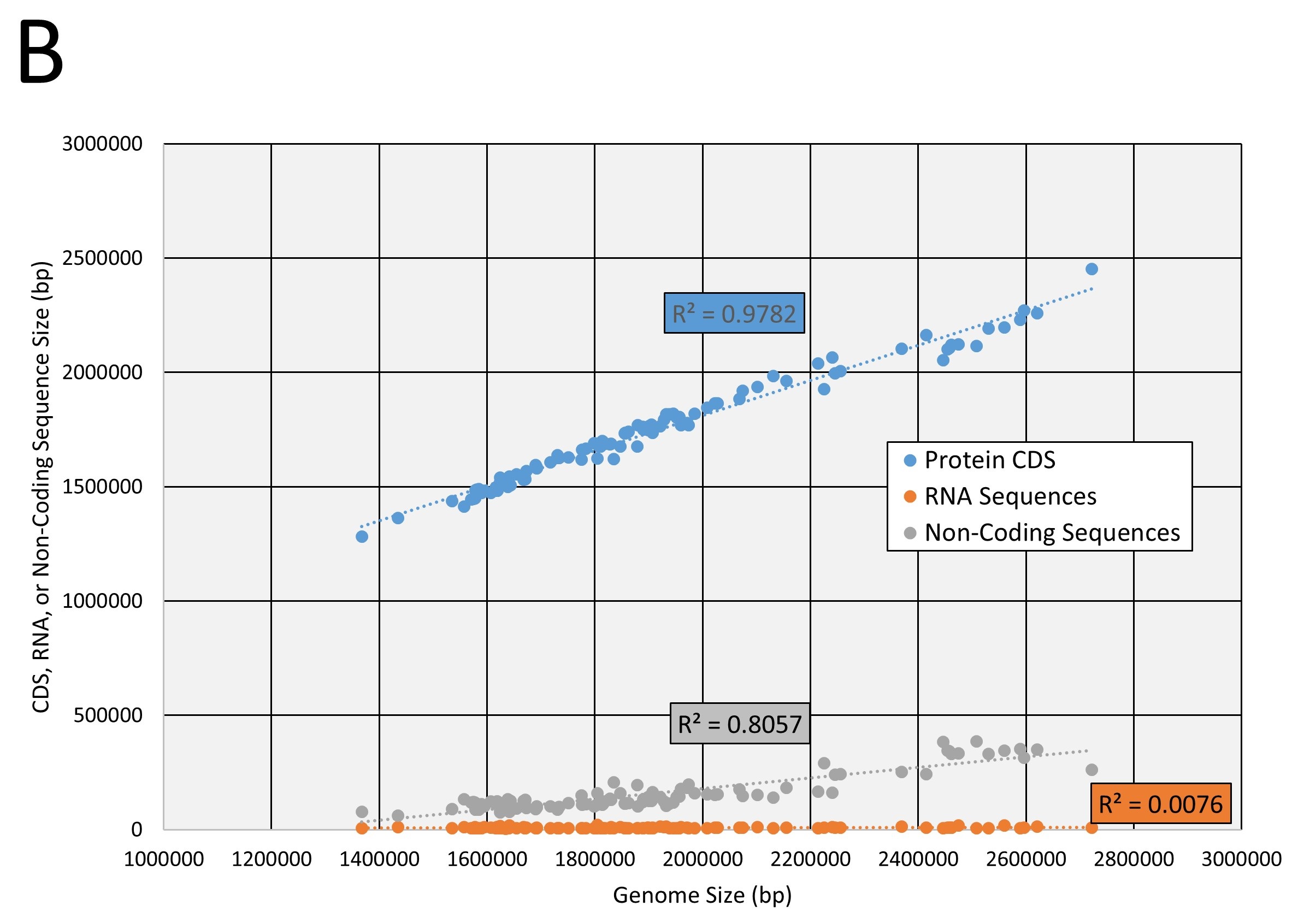

Supplement: Supplementary file 7 — Figure S1. A) Genome sizes were plotted against the number of annotated protein coding sequences (CDS) and GC content. For all Helicobacter genomes, a linear relationship existed for genome size versus number of annotated protein CDS (R2 = 0.8939). Three EHS genomes (H. muridarum ST1, H. pametensis ATCC 51478, and H. cholecystus ATCC 700242) and one gastric genome (H. bizzozeronii CCUG 35545) appeared to be outliers and are indicated in the graph. Diamonds (◆), gastric genomes; circles (●) EHS genomes; cross (X), C. jejuni genome. B) Genome sizes were plotted against the additive size of all protein CDS, RNA genes, and non-coding gene sequences. Linear relationships existed for genome size versus size of protein CDS (R2 = 0.9782) and non-coding gene sequences (R2 = 0.8057), but not for RNA gene sequences (R2 = 0.0076). (ZIP 458 kb) [file 12864_2018_5171_MOESM7_ESM.zip › Slide10-1.jpg]

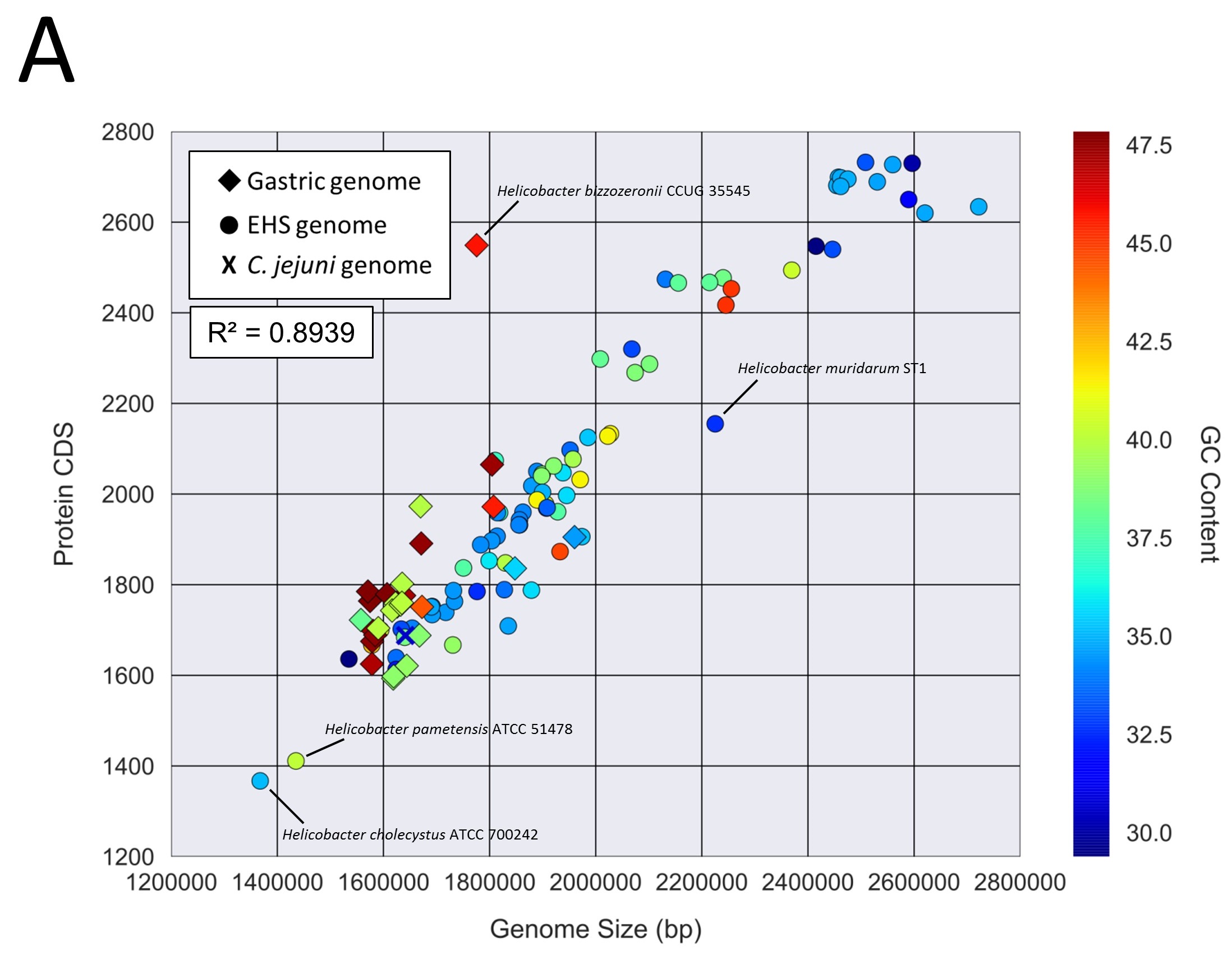

Supplement: Supplementary file 7 — Figure S1. A) Genome sizes were plotted against the number of annotated protein coding sequences (CDS) and GC content. For all Helicobacter genomes, a linear relationship existed for genome size versus number of annotated protein CDS (R2 = 0.8939). Three EHS genomes (H. muridarum ST1, H. pametensis ATCC 51478, and H. cholecystus ATCC 700242) and one gastric genome (H. bizzozeronii CCUG 35545) appeared to be outliers and are indicated in the graph. Diamonds (◆), gastric genomes; circles (●) EHS genomes; cross (X), C. jejuni genome. B) Genome sizes were plotted against the additive size of all protein CDS, RNA genes, and non-coding gene sequences. Linear relationships existed for genome size versus size of protein CDS (R2 = 0.9782) and non-coding gene sequences (R2 = 0.8057), but not for RNA gene sequences (R2 = 0.0076). (ZIP 458 kb) [file 12864_2018_5171_MOESM7_ESM.zip › Slide9-1.jpg]
